# The Phylogeography of Potato Virus X Shows the Fingerprints of Its Human Vector

**DOI:** 10.3390/v13040644

**Published:** 2021-04-09

**Authors:** Segundo Fuentes, Adrian J. Gibbs, Mohammad Hajizadeh, Ana Perez, Ian P. Adams, Cesar E. Fribourg, Jan Kreuze, Adrian Fox, Neil Boonham, Roger A. C. Jones

**Affiliations:** 1 Crop and System Sciences Division, International Potato Center, La Molina Lima 15023, Peru; s.fuentes@cgiar.org (S.F.); a.perez@cgiar.org (A.P.); j.kreuze@cgiar.org (J.K.); 2 Emeritus Faculty, Australian National University, Canberra, ACT 2600, Australia; adrian_j_gibbs@hotmail.com; 3 Plant Protection Department, Faculty of Agriculture, University of Kurdistan, Sanandaj 6617715175, Iran; m.hajizadeh@uok.ac.ir; 4 Fera Science Ltd., Sand Hutton York YO41 1LZ, UK; Ian.Adams@fera.co.uk (I.P.A.); Adrian.Fox@fera.co.uk (A.F.); 5 Departamento de Fitopatologia, Universidad Nacional Agraria, La Molina Lima 12056, Peru; cfribourgs@hotmail.com; 6 Institute for Agrifood Research Innovations, Newcastle University, Newcastle upon Tyne NE1 7RU, UK; Neil.Boonham@newcastle.ac.uk; 7 UWA Institute of Agriculture, University of Western Australia, 35 Stirling Highway, Crawley, WA 6009, Australia

**Keywords:** potato, virus disease, potato virus X, South America, Andean crop domestication center, strain groups, high-throughput sequencing, phylogenetics, population genetics, Andean lineages, dating, interpretation, evolution, prehistory, biosecurity significance

## Abstract

Potato virus X (PVX) occurs worldwide and causes an important potato disease. Complete PVX genomes were obtained from 326 new isolates from Peru, which is within the potato crop′s main domestication center, 10 from historical PVX isolates from the Andes (Bolivia, Peru) or Europe (UK), and three from Africa (Burundi). Concatenated open reading frames (ORFs) from these genomes plus 49 published genomic sequences were analyzed. Only 18 of them were recombinants, 17 of them Peruvian. A phylogeny of the non-recombinant sequences found two major (I, II) and five minor (I-1, I-2, II-1, II-2, II-3) phylogroups, which included 12 statistically supported clusters. Analysis of 488 coat protein (CP) gene sequences, including 128 published previously, gave a completely congruent phylogeny. Among the minor phylogroups, I-2 and II-3 only contained Andean isolates, I-1 and II-2 were of both Andean and other isolates, but all of the three II-1 isolates were European. I-1, I-2, II-1 and II-2 all contained biologically typed isolates. Population genetic and dating analyses indicated that PVX emerged after potato’s domestication 9000 years ago and was transported to Europe after the 15th century. Major clusters A–D probably resulted from expansions that occurred soon after the potato late-blight pandemic of the mid-19th century. Genetic comparisons of the PVX populations of different Peruvian Departments found similarities between those linked by local transport of seed potato tubers for summer rain-watered highland crops, and those linked to winter-irrigated crops in nearby coastal Departments. Comparisons also showed that, although the Andean PVX population was diverse and evolving neutrally, its spread to Europe and then elsewhere involved population expansion. PVX forms a basal *Potexvirus* genus lineage but its immediate progenitor is unknown. Establishing whether PVX′s entirely Andean phylogroups I-2 and II-3 and its Andean recombinants threaten potato production elsewhere requires future biological studies.

## 1. Introduction

Potato virus X (PVX, genus *Potexvirus*, family *Alphaflexiviridae*) [1,2] was one of the first potato (*Solanum tuberosum*) viruses described [3,4,5,6]. It is one of over 50 viruses now found infecting potato crops around the world and, historically, has been the subject of much research [7,8,9,10,11,12,13]. It is spread by contact between healthy and infected foliage or roots of potato, tobacco and tomato plants. It also spreads by contact when PVX-contaminated machinery moves through potato crops and from tuber-to-tuber when potato tubers are cut with PVX-contaminated knives before planting. No specific PVX vectors have been found despite a wide range of invertebrate species being tested, and it is not transmitted via true seeds of infected potato plants. However, non-specific transmission by biting insects has been reported [13]. Long-distance spread within regions or worldwide depends on the movement of virus-infected plant materials, usually in the trade of infected seed potato tubers [7,8,9,10,11,12,13,14]. PVX strains differ in virulence, mostly causing mild leaf mosaic symptoms, but severe strains cause obvious mosaics [3,6,7,8,13,15,16]. PVX infection usually depresses the yield of potato tubers by 5–20%, but up to 40% with severe PVX strains [8,9,10,11,12,13,17,18]. In potato plants, mixed infections of PVX with potato virus A (PVA) or potato virus Y (PVY), both potyviruses (family, *Potyvirideae*), causes the classic severe foliage diseases ‘crinkle’ (PVX + PVA) and ‘rugose mosaic’ (PVX + PVY), and much greater tuber yield losses [8,13,19,20]. Mixed infection of PVX with potato virus S (PVS; genus, *Carlavirus*, family, *Betaflexiviridae*) also increases the severity of potato foliage symptoms [21].

PVX infects herbaceous dicotyledonous plants, especially those belonging to the *Solanaceae*, and has filamentous virions 470–580 nm long, each of which contains a single positive-sense ssRNA genome (c. 6400 nt) and around 1300 copies of the coat protein (CP)—8.9 CP units per helix turn [22]. The genome has five open reading frames (ORFs). The first of these ORFs encodes a complex RNA-dependent RNA polymerase [23], followed by the three overlapping genes that encode the component proteins of the triple gene block (TGB1-3) cell-to-cell movement protein, and, at the 3′ end is the gene for the CP. Cockerham [24] used potato cultivar differentials with two PVX hypersensitivity genes, *Nx* and *Nb*, to classify PVX strains biologically. Strain group 1 (strain group = pathotype) isolates fail to avoid detection by either of these hypersensitivity genes, so a hypersensitive resistance (HR) phenotype develops with both of them, strain group 2 overcomes *Nx,* but HR occurs with *Nb*, strain group 3 overcomes *Nb,* but HR occurs with *Nx*, and strain group 4 overcomes both genes, so a susceptible phenotype always develops unless extreme resistance (ER) gene *Rx* is also present [25,26,27]. Before genes *Nx* and *Nb* were exploited in potato breeding programs, and healthy seed potato production schemes became more sophisticated, strain group 1 was commonly found in mixtures with strain groups 2 or 3 [15,16,24]. In Europe, more recently, strain group 3 isolates became the most abundant, whereas strain groups 1 and 2 became rare, while strain group 4 isolates are less competitive and so are rarely found in the field [28,29]. All four PVX strain groups were found infecting potato crops in the Andean region of South America [30,31,32]. Neither *Nx* nor *Nb* are temperature-sensitive [33]. PVX strain group 4 isolates that overcome not only *Nx* and *Nb* but also *Rx* have been reported on two occasions, Bolivian isolate X^HB^ and Argentinian isolate X^MS^ [31,34]. Jones [35,36] described selection of PVX strain group 4 isolates from strain groups 2 or 3. PVX′s coat protein (CP) gene elicits an HR phenotype in potato plants with gene *Nx* and an ER phenotype with gene *Rx* [37,38,39], whereas its movement protein (MP) does this with gene *Nb* [40]. A single amino acid (aa) change in the CP or MP gene was sufficient to change the phenotype from HR to susceptible in potato cultivars with genes *Nx* and *Nb,* respectively. This was also so with the CP of isolate X^HB^ and gene *Rx,* an ER phenotype being altered to a susceptible one [37,40,41].

When Cox and Jones [42] compared the CP gene nt sequences of 13 new PVX isolates from Australia or the UK with those of 72 others from GenBank, phylogenetic analysis revealed two major phylogroups (I and II) and two minor phylogroups (II-1 and II-2). Most isolates were in major phylogroup I, and these came from Australasia, Africa, Asia, Europe, South America (non-Andean) and North America, and they included Argentinian isolate X^MS^. Isolates in minor phylogroup II-1 were from Europe, but those in II-2 were from the Andean region of South America or North America, and these included isolate X^HB^. Isolates from strain groups 1, 3 and 4 were in major phylogroup I, whereas isolates in strain groups 2 and 4 were present in II-1 and II-2. Therefore, as strain group 4 isolates were in both major phylogroups, no direct correlation existed between phylogenetic placement and biological strain groups. When Kutnjak et al. [43] compared the complete genomes of nine Peruvian PVX isolates with those of 20 complete PVX genomes, phylogenetic analysis revealed that two were within minor phylogroup II-2 together with previously reported Andean isolates. However, six others were all in an entirely new Andean minor phylogroup they called II-3, and one was in major phylogroup I. They found no evidence of PVX recombination events. Phylogenetic analysis of all available CP sequences placed two other Andean isolates from Colombia within II-3, suggesting it might be widespread in the Andean region. Subsequently, phylogenetic analysis using CP sequences of three further Colombian isolates placed these in major phylogroup I [44,45].

About 9000 years ago, potato was domesticated from its wild potato ancestors in the Altiplano regions of Peru and Bolivia in the South American Andes mountains [46]. After the 1542 arrival of Europeans to the Americas, potato land races (=native potato cultivars) were taken to Europe during the Columbian Exchange of animals and plants between the Americas and Eurasia [47] and introduced from there to other continents [48]. During early studies in which collections of Andean potato land races and wild potato species were tested for presence of common potato viruses, PVX was often the most frequently detected [49,50,51,52,53,54]. Widespread occurrence of PVX was also revealed by studies in Peru of Andean potato germplasm collections and leaf samples collected from land races and locally bred modern potato cultivars growing in the field [30,31,55]. This common occurrence of PVX was accompanied by presence of PVX resistance genes *N*x, *Nb* and *Rx* in potato land races and wild potato species [26,27,32,56,57].

Recently, we have reported the properties of genomic sequences of isolates of three common potato viruses (PVA, PVS and PVY), obtained from potato land races or locally bred modern potato cultivars growing in the Peruvian Andean potato domestication center, compared them with isolates from other world regions, and made deductions concerning their evolution [58,59,60]. In this paper, we report a similar study of PVX. The results of these analyses provide new information on the phylogenetics and population genetics of PVX. They also greatly enhance our understanding of the origins and spread of this virus by humankind.

## 2. Materials and Methods

### 2.1. Virus Isolates

The 11 historical isolates, all but one of which (DX) were sequenced, were collected between 1940 and 1985 and came from Peru (A, CP, DP, E), Bolivia (HB) and the UK (B, DX, EX), or were strain group 4 isolates derived from three of them (CP4, DX4, EX4) (Table 1a). Three Peruvian isolates (A, DP, E) were kept in desiccated leaf tissue over silica gel at 4 °C at the National Agrarian University, La Molina, Lima, Peru, before being sent to the UK for sequencing in 2018. All other historical isolates were maintained in a collection of historic freeze-dried virus isolates kept at FERA Science Ltd., York, UK. In earlier studies, all these isolates had been inoculated to potato cultivar differentials to establish which strain groups they belonged to [24,28,30,31,35,36].

**Figure 1:**
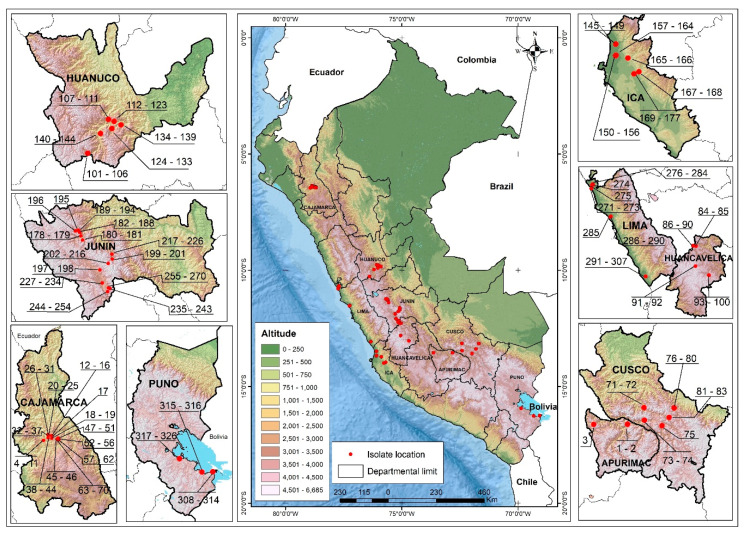
Map of sample collection sites in the Andean Highlands showing where potato leaf samples were obtained. Peru’s Andean highlands are shown as brown, the country′s coastal desert and Amazonian jungle regions as green and surrounding countries as white. The red dots marked on the main map represent the locations sampled, and the names marked on it are those of the countries’ regional departments sampled (black lines are departmental boundaries). The red dots marked on the individual department maps clustered on either side show each collection site, and the numbers indicate each individual infected sample collected. Individual collection sites are numbered (Supplementary Materials (SM) File S1, column C). The names of the Departments also provide the first three letters of each isolate name (SM File S1, column F).

Three hundred and twenty-six new PVX isolates from Peru were obtained from 269 leaf samples derived from 994 individual potato plants collected between 2016 and 2018 in the northern, central and southern Andean highlands of Peru (Figure 1), and some samples were infected with more than one PVX variant. The potato plants sampled showed foliage symptoms indicating virus infection. They came from nine Peruvian Departments (i.e., different administrative regions of Peru) as follows (number of sequences/number of samples): north: Cajamarca (67/60), center: Huanuco (44/37), Junin (93/77), Huancavelica (17/15), Lima (37/29), Ica (33/26), and south: Apurimac (3/3), Cusco (13/10), Puno (19/12). Each sample was placed in a separate labelled paper filter bag, nine of which were placed together in a zip-lock plastic bag with 100 g of dehydrated silica gel for rapid desiccation. The silica gel was changed after 24–48 h and the combined samples taken to the International Potato Center (in Spanish = Centro Internacional de la Papa, CIP) in Lima for processing. For the 326 new Peruvian isolates, Table 1b and Supplementary Materials (SM) Table S1 show which Department each came from, the year it was isolated and the total number of samples and isolates sequenced. A simplified searchable spread-sheet version (SM File S1) provides more detail of the provenance of each isolate and, where available, the potato cultivar from which it was isolated, and, for each Peruvian isolate, the number of the site from which it was collected, as shown in Figure 1. Each isolate name starts with a three-letter mnemonic of the Department where it was collected.

In addition, three new PVX isolates from Burundi, East Africa, were collected from potato (*S. tuberosum* subsp. *tuberosum*) cv. Ndinamagara (=Cruza 148) in 2016 at Kanyunya (JEO11-14, MT520806), Rwibaga (JEO11-25, MT520804) and Nyamugari (JEO11-30, MT520805), Bujumbura Rural Province. 

The 49 PVX sequences already available in GenBank were downloaded in July 2020.

### 2.2. High-Throughput Sequencing 

In the UK, samples of freeze-dried PVX-infected leaf material containing one each of 11 isolates (A, CP, CP4, DP, E, HB, B, DX, DX4, EX, EX4) were subjected to high-throughput sequencing (HTS) in 2016–2019 (Table 1a). Total RNA was extracted from each sample using the Total RNA kit (Qiagen, UK), including the optional DNase treatment. An indexed sequencing library was produced from the total RNA using a Scriptseq complete plant leaf kit (Illumina, USA) and sequenced on a MiSeq instrument (Illumina), using a 600 cycle V3 kit. The methods followed are described in more detail by Fox et al. [61]. Ten isolates provided a complete PVX ORF. No other virus sequences were associated with these complete ORFs and no sequence of isolate DX was obtained. The new genomic sequences with complete ORFs were mostly c. 6435 nts long. Their final sequences were submitted to GenBank with Accession Codes MT708134-MT708143 (Table 1a and SM File S1). 

In Peru, total RNA was extracted from each potato leaf sample (Table 1b) using trizol, as instructed by the manufacturer. The large RNA fraction was precipitated by adding an equal volume of 4M LiCl at ~4 °C (on ice) overnight, followed by centrifugation. The remaining small RNA fraction was subsequently precipitated by adding one volume of isopropanol followed by centrifugation. Small RNAs were separated on 3.5% agarose gels and bands corresponding to ~20–25 nts excised and purified using quantum prep freeze and squeeze columns (BioRad). Small RNA libraries were prepared using the protocol of Chen et al. [62] and sent for sequencing on a HiSeq4000 by a commercial provider (Fasteris Life Sciences SA, Switzerland). Small RNA sequences were analyzed using VirusDetect v1.6 [63] to identify all viruses infecting the plants, and samples in which PVX was detected were selected for further analysis. Using the Geneious R11.1.3 software package (https://www.geneious.com; accessed on 1 May 2019), the PVX contigs produced by VirusDetect were extracted for each positive sample and a consensus was generated. The small RNAs were mapped back to the consensus to confirm the quality of the assemblies and make any corrections as necessary. Their final sequences are recorded in GenBank and have Acc Codes MT752611–MT752936 (SM Table S1 and SM File S1). The three Burundi leaf samples were desiccated in silica gel, similar to the Peruvian samples which were sent under license to Peru where they were sequenced with the Peruvian samples. Their final sequences were submitted to GenBank with Accession Codes MT520804–MT520806 (SM File S1). All the new genomic sequences with complete ORFs were c. 6450 nts long.

### 2.3. Sequence Analysis

Genomic sequences were edited using BioEdit [64] to extract their five gene regions (replicase, gp2 (25K), gp3 (12K), gp4 (8K) and gp5 (CP)). The sequences of each gene region were aligned using the encoded aa′s as a guide, by the TranslatorX online server [65] (http://translatorx.co.uk; accessed on 1 June 2019) with its Multiple Alignment using Fast Fourier Transform (MAFFT) option [66]. The alignments were appended sequentially to form an alignment of concatenates with all genes in the same reading frame. A separate CP alignment was made from the new CP genes after 45 near-duplicate Peruvian sequences had been removed for computing convenience, and all of the PVX CP genes downloaded from GenBank.

The concatenated sequences (concats) were tested for the presence of phylogenetic anomalies using the full suite of options in the Recombinant Detection Program RDP4 [67] with default parameters [68,69,70,71,72,73,74,75,76,77]. Anomalies found by less than five methods and with greater than 10^−5^ random probability were ignored. Models for Maximum Likelihood (ML) analysis were compared using MEGA7 [78]. The best-fit models were found to be GTR + Г_4_ + I [79] for nucleotide (nt) sequences and LG + Г_4_ + I [80] for aa sequences. 

Phylogenetic trees were calculated using the neighbor joining (NJ) option in ClustalX [81], and/or in Phylogenetic Maximum Likelihood (PhyML) 3.0 for ML [82]. In PhyML, the statistical support for their topologies was assessed using the Shimodaira and Hasegawa (SH) method [83]. Trees were drawn using Figtree Version 1.3 (http://tree.bio.ed.ac.uk/software/figtree/; accessed on 12 May 2018) and a commercial graphics package. PATRISTIC [84] was used to check for mutational saturation by comparing the patristic distances of the nt phylogenies with those of the aa′s they encoded and confirmed by the method of Xia [85]. The BlastN and BlastP online facilities of GenBank [86] were used to search for potexvirus sequences with which to compare, and also to root, the PVX phylogenies.

The program DnaSP v.6.10.01 [87] was used to analyze genetic differences between selected populations of sequences. We used it to estimate average pairwise nt diversity (π), number of synonymous sites (SS), number of non-synonymous sites (NS), mean synonymous substitutions per synonymous site (dS), mean non-synonymous substitutions per non-synonymous site (dN) and ratio of non-synonymous nt diversity to synonymous nt diversity (dN/dS). It was concluded that genes were under positive, neutral or negative selection when their dN/dS ratios were >1, =1 and <1, respectively. Tajima′s D statistical test was used to identify non-random evolutionary events such as population expansion, bottlenecks and selection by comparing the estimated number of segregating sites with the mean pairwise difference among sequences [88]. DnaSP v.6.10.01 was also used to assess the extent of genetic differentiation of PVX populations, measured as the amount of gene flow between them. This was done using the coefficient of genetic differentiation F_ST_ (=the inter-populational component of genetic variation or the standardized variance in allele frequencies across populations) [89] and the gene flow parameter Nm (the product of the effective population number and rate of migration among populations) [90].

The TempEst program [91] was used to check for the presence of a linear temporal signal in all the dated sequences, and all those in Cluster B. The ‘Least Squares Dating’ (LSD) method Version lsd-0.3beta of To et al. [92] was used to estimate the TMRCAs (Time to the Most Recent Common Ancestor) of Cluster B. The statistical significance of correlation coefficients was calculated using the Social Science Statistics online site (https://www.socscistatistics.com/pvalues/pearsondistribution.aspx; accessed on 3 August 2020). Some alignments were separated into three sub-alignments using NSplitter (https://github.com/HarryGibbs/NSplitter; accessed on 3 August 2020): one was of all the codon positions that had only changed synonymously, another was of codons that included at least one non-synonymous change and the third was of codons that had not changed. 

## 3. Results

### 3.1. Sequence Alignments

The 388 genomic sequences (339 new and 49 downloaded from GenBank) were edited and converted as described above to an alignment of concats 6357 nts long. A separate alignment of 488 CP genes was made from the CP genes of the new sequences after 45 near-duplicates were removed as described above and 128 CP sequences from GenBank were added. Three quarters of the CP sequences were 711 nts long, but 119 of the Peruvian sequences were 720 nts long, and three from the UK (the EX-2, B and EX sequences; GU384737, GU384738 and X88782) were 744 nts long with all the inserted codons being situated around 16 codons from their N-termini. 

### 3.2. Recombination Analyses

When the concat sequences were checked for phylogenetic anomalies using Recombination Detection Program No. 4 (RDP4), 18 of the sequences, 17 of them from Peru, were found to have recombinant (rec) regions (Table 2). The rec sequence not from Peru was HQ450387 from the USA. Peruvian rec sequence M31541 had an Argentinian major parent (X55802) and an unknown minor parent. The 18 rec sequences were removed from the alignment used for phylogenetic and population genetic analysis because rec sequences distort the results of most algorithms used for reconstructing phylogenies. The CP genes were also checked by RDP4, but no additional rec sequences were found. Thus, a significantly smaller proportion of the PVX population was recombinant compared with, for example, the population of PVY, where around 41% of isolates were recombinant [59].

### 3.3. Phylogroups

**Figure 2:**
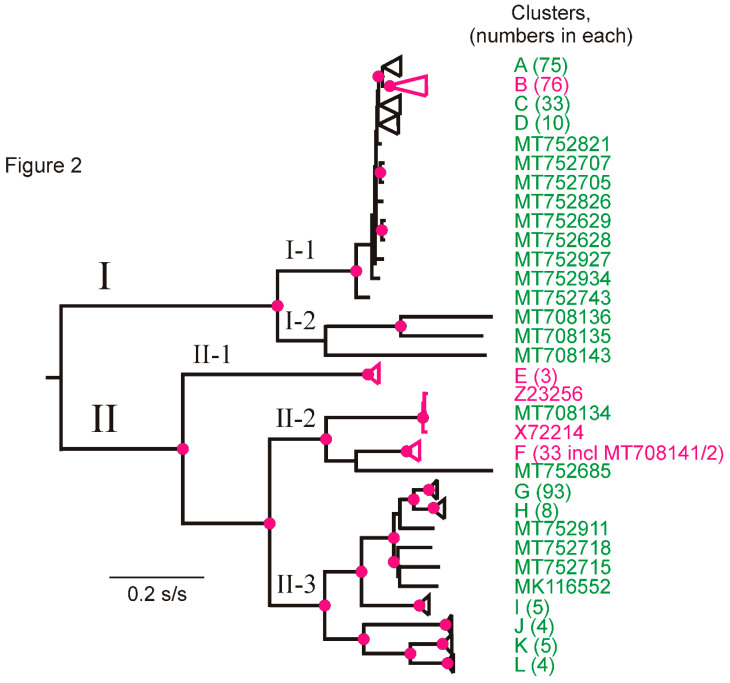
A Maximum Likelihood phylogeny of the 370 non-rec potato virus X concats. The phylogroups and minor phylogroups have Latin-Arabic numbers, and the clusters (SM File S2) have a capital letter, and in brackets next to them, the number of isolates within each. The Accession Codes of singletons are shown. Singletons or clusters of isolates only from South America, mostly Peru, are green, whereas singletons from other regions of the world, or clusters containing such isolates, are in red. All details are given in SM File S1. Red disks mark the nodes with >0.95 SH support. Scale bar: s/s means substitutions/site. SM File S2 shows the Accession Codes of the isolates in the different clusters shown in this figure.

A ML phylogeny (Figure 2) was generated from the non-rec concats using PhyML [82]. The topology of the phylogeny was the same as that reported for PVX by Cox and Jones [42] and Kutnjak et al. [43], who used 85 CP sequences and 29 complete genomes respectively, and two different methods of tree building: ML and NJ. Their phylogenies had a basal divergence, which produced two major phylogroups (I and II) that separated into five minor phylogroups, and, in conformity with the earlier reports, we call these I-1, I-2, II-1, II-2 and II-3. All phylogroup 1 concat isolates found previously were placed in minor phylogroup I-1, and I-2 only comprised three newly sequenced historical Peruvian isolates. We also grouped the distal parts of the phylogeny into 12 statistically supported clusters, A–L (Figure 2), with 20 singletons. The sequences in each of the clusters are recorded in SM File S2 with the details of each isolate in SM File S1. The topology of the ML phylogeny of CP sequences was closely similar to that of the concats (data not shown). However, it had less well-defined clusters and less statistical support: 19.9% of the nodes of the concat tree were fully supported (SH = 1.0, [83]), whereas only 0.6% of the CP tree nodes were fully supported, and similarly, 22.1% and 14.8% respectively, of the other nodes had SH support values of 0.90–99. Nonetheless, the CP data adds detail to the distribution of Andean and non-Andean isolates in the PVX phylogeny and shows that whereas only Andean isolates form phylogroups I-2 (3 sequences) and II-3 (213 sequences), all the other phylogroups contain both Andean and non-Andean sequences, and, likewise, in phylogroup I-1, cluster D and the singletons are exclusively Peruvian, whereas clusters A, B and C include both Andean and non-Andean sequences. This distribution indicates that PVX originated in or near Peru and spread from there to the remainder of the world.

The cluster B concats were 76, half from Peru, three from Colombia and the remainder from other continents: one of its two largest subclusters was of 29 concats only from Peru (SH support 0.85), and the other of 35 isolates (SH support 1.0) included 12 from Peru together with isolates from Colombia (1) in the Andean region, and China (2), India (1), Iran (2), Japan (5), Korea (1), Netherlands (1), Russia (1), Switzerland (1), Taiwan (1), Tunisia (2), UK (3) and USA (2). This cluster was examined in more detail—see ‘dating’ Section 3.6 below.

Among the historical PVX isolates belonging to known biological strain groups, the phylogenetic placement of the new complete genomic sequences (Table 1a) was as follows: isolates B (MT708140) from Scotland, and EX (MT708138) and EX4 (MT708137) from England, which belong to strain groups 2 or 4, were in minor phylogroup II-1, isolates HB (MT708134) from Bolivia, and both CP (MT708142) and CP4 (MT708141) from Peru, which belong to strain groups 2 or 4, were in minor phylogroup II-2, isolates A (MT708136), DP (MT708143) and E (MT708135) from Peru, which belong to strain groups 1 or 3, were in minor phylogroup I-2, and isolate DX4 (MT708139) from England, which belongs to strain group 4, was in minor phylogroup I-1.

The PVX sequences were separated into five concat minor phylogroups (Figure 2) and analyzed using DnaSP 6. The complete concat sequences within each of them were analyzed, as were their five individual genes (SM Table S2): the I-2 and II-1 minor phylogroups were of only three sequences each, whereas the I-1, II-2 and II-3 minor phylogroups were represented by 203, 37 and 123 sequences, respectively. The genetic diversity estimates (π) confirmed that minor phylogroups I-2 and II-3, which contained only Andean isolates, are more genetically diverse than the other PVX minor phylogroups, as were most of their individual genes, except TGB3. The RdRp and the TGB3 genes, the largest (69% of the concat) and smallest (3.3%) respectively, are the most variable PVX genes. Furthermore, as for most virus genomes, the dN/dS ratios of the concats and all genes are less than one (mean 0.081), indicating that they have been under strong purifying (negative) selection. The smallest dN/dS ratio (mean 0.070) was that of the CP gene, perhaps because of its many functions: the activation of PVX RNA translation [93], the transport of infection [94] and viral genome RNA encapsidation [95].

**Figure 3:**
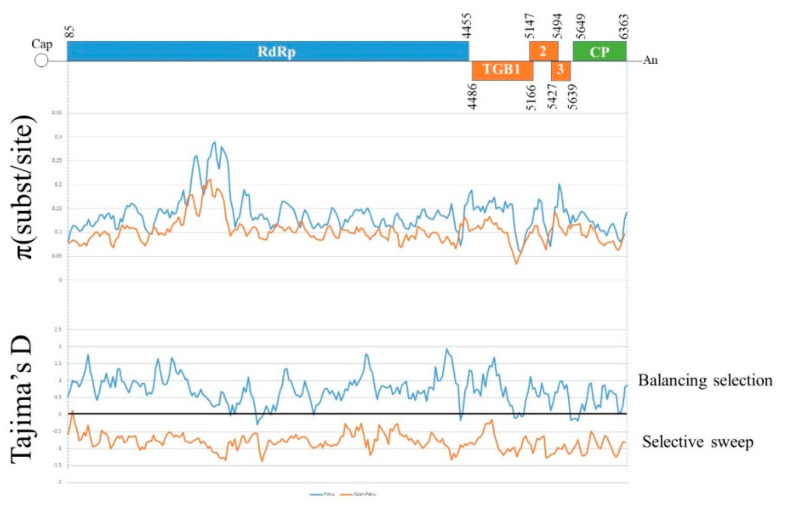
The mean genetic diversity of the aligned concats from 322 (Peruvian, blue) and 47 (non-Peruvian, red) potato virus X genomes. Estimates of π (substitution/sites) and of Tajima′s D metric were made in a window of 100 nts with a step of 25 nts.

Tajima′s D statistical test [88] distinguishes which gene sequences have been evolving randomly (‘neutrally’) from those that have been evolving under non-random processes, such as selection, demographic expansion or contraction. This test returns a negative value when there are more polymorphisms in the population than expected under neutral processes and calculates the probability that the result is significant. We applied this test to the concat and individual genes of the three best-represented minor phylogroups and found that the concats and most of the individual gene sequences of I-1 and II-2, but not II-3, returned significant negative values (SM Table S2). This difference therefore correlates with the fact that I-1 and II-2, but not II-3, included isolates from outside the Andes, and indicates that the non-Andean populations of PVX were established by population expansion of migrants from the Andean population. The Tajima′s D difference between Andean and non-Andean concats is consistent throughout their length in all genes, as was confirmed using the sliding window function of DnaSP 6 (Figure 3).

The details of the phylogeny of Cluster B (SM Figure S1), however, indicate that the spread of PVX from its Andean population to other parts of the world was not a simple single-direction migration and divergence. Several distinct subclusters within Cluster B include isolates from both the Andes and elsewhere (SM Figure S1; green and red Accession Codes, respectively). If, for example, one applies cladistic reasoning to sub-cluster B1 of SM Figure S1, it has a clear Peruvian origin as its basal divergences all involve Peruvian isolates, whereas, by the same reasoning, sub-cluster B2 clearly originated from outside the Andes but has recently spread to Peru. Thus, it seems that although PVX has mostly spread from the Andes to other parts of the world, it may have done so on more than one occasion, and there has also been some complex ‘repatriations’. 

### 3.4. World Populations of PVX

DnaSP 6 was used to assess the genetic linkage or ‘gene flow’ between PVX populations from different regions of the world. This was measured using F_ST_ and the ‘gene flow parameter’ (Nm), which indicate maximal linkage when F_ST_ is the smallest positive value, and Nm the largest positive value [96,97]. It can be seen (Table 3a) that the Andean PVX population (*n* = 346) is linked most closely with the European population (*n* = 17) by both the F_ST_ metric (0.073, the smallest), and by the Nm metric (3.19, the largest). Also, although similar values of the two measures were obtained for the Africa:Asia comparisons, these are less reliable as there were only six sequences in the Africa group. The comparisons of the only three North American concats were omitted as they gave negative metrics.

F_ST_ values were also calculated from the alignment of CP sequences grouped into ‘continent’ populations (SM File S1). The results (Table 3b) show that the PVX populations of each of the ‘continents’ are primarily linked with that of West Eurasia (Europe plus Russia and Middle East): the smallest F_ST_ for comparisons involving East Asia (China, Korea and Japan) is West Eurasia (0.041), for one involving the Indian Subcontinent (India/Pakistan/Bangladesh) is also West Eurasia (0.069), and likewise for the Andean region (0.205). Thus, a combination of Tajima′s D, F_ST_ and Nm analyses of PVX gene populations indicate that PVX most likely spread first from the Andes to Europe/Russia/Middle East and from there, separately, to East Asia and the Indian subcontinent.

### 3.5. PVX Populations of Peru

**Figure 4:**
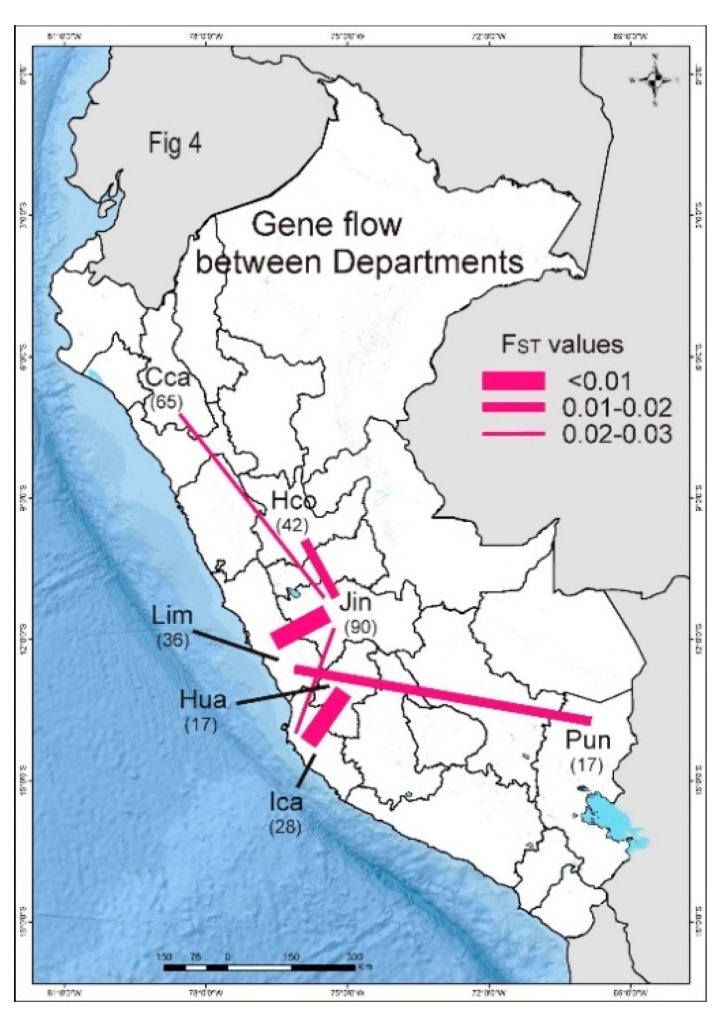
Cartoon showing the most significant genetic linkages between the potato virus X populations of different Peruvian Departments. Linkages are indicated by their F_ST_ values [97,98].

The populations of PVX isolated from different Departments of Peru (Figure 1) were compared. The Peruvian population is apparently “well mixed” [98] as there was no correlation between the phylogenetic clusters and the Peruvian Departments from which samples were collected. No cluster, however small, had sequences from a single site, the more sequences in a phylogenetic cluster the greater the number of Departments in which it was found (Figure 4). The smallest clusters were found in two to four Departments that were not necessarily adjacent, and the largest cluster of isolates (G) was found in all nine Departments, and included 14 sequences from Cajamarca in North Peru and four from Puno in South Peru at the border with Bolivia, where the northern portion of Lake Titicaca is situated (note: the distance between Cajamarca and Lake Titicaca is more than 1500 km). 

**Figure 5:**
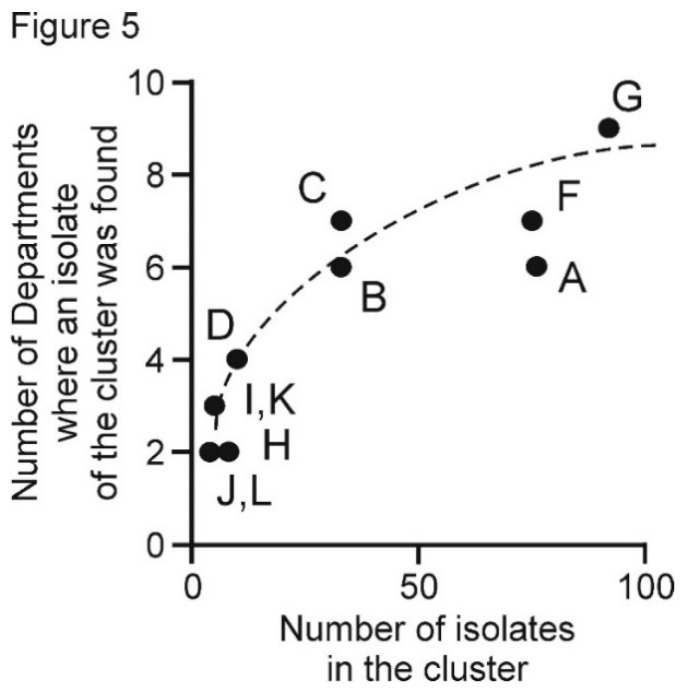
Graph of the number of Peruvian Departments in which an isolate of each potato virus X cluster was found, plotted against the number of isolates in each cluster. Letters A–L stand for the clusters A–L in Table 2 and Figure 2. As there were no Peruvian isolates within cluster E, it is not included in this Figure.

Gene flow between Department populations was assessed using the F_ST_ measure [89,97,98]. The six smallest positive F_ST_ values of the 36 pairwise comparisons (Table 4) are summarized graphically in Figure 5. They were least for the Junin:Lima (0.009) and Huancavelica:Ica (0.004) comparisons, intermediate for the Lima:Puno (0.012) and Huanuco:Junin (0.016) comparisons and the Cajamarca:Junin (0.021) and Ica:Junin (0.027) comparisons gave the largest F_ST_ values. All the other 30 comparisons yielded even larger positive or negative F_ST_ values and included all of the comparisons involving the two least sampled populations: 3 samples from Apurimac, and 12 from Cusco. Thus, the most significant PVX gene flow (i.e., genetic linkage) was between: (i) the mountain Departments of Cajamarca, Huanuco, Junin, Huancavelica and Puno, where potatoes are grown under natural rainfall in summer, and (ii) the coastal Departments of Ica and Lima, where potatoes are grown under irrigation in winter, and the mountain Departments closest to them (Junin for Lima, Huancavelica for Ica), which provide the seed potatoes for their crops.

**Figure 6:**
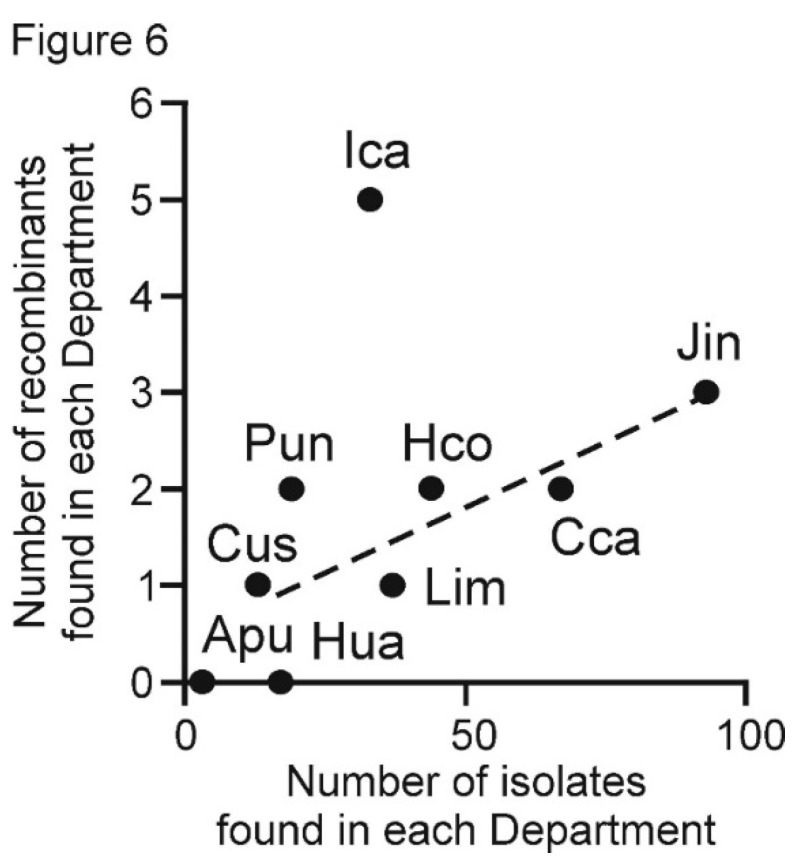
Graph of the number of recombinants found in each Department plotted against the total number of potato virus X isolates collected from each Department.

Although only 18 rec concats (4.9% of the 388) were found (Table 2), 17 of these were Peruvian. One of these had a major Argentinian parent. The 16 with entirely Peruvian parents support the conclusions of the F_ST_ comparisons of the PVX populations from different Departments. First, they confirm that the Peruvian PVX population is geographically “well mixed” as the number of rec sequences found in each Department was broadly related to the total number of isolates collected from that Department (Figure 6), with the exception of the Ica population, which had twice as many rec sequences as any other Department yet was of average size. The distribution of the likely ‘parental’ isolates identified by the RDP4 analysis was not obviously related to their phylogeny or sampling density. Table 2 also shows that only four of the rec sequences were isolated from the same Department. This is because both of their ‘parental’ isolates involved just three rec sequences and parents from Junin and one from Ica, and only two more were from the same Department as the major ‘parent’ (Ica and Puno). In addition, the other nine were isolated from plants collected from a Department that did not provide either ‘parent’. Most ‘parental’ isolates, both major and minor, were from Junin (ten and eight, respectively) or from Ica (three and five, respectively), and only four from other Departments. Thus, the provenance and parentage of the rec sequences again supports the conclusion that there has been much movement of PVX between the coastal Departments and the mountain Departments that supply most of the seed potatoes for their winter-irrigated crops, namely between Lima and Ica, and between Junin and Huancavelica.

### 3.6. Dating

The ‘collection date’ of the 370 non-rec PVX isolates supplying the concats is known (SM File S1). Therefore, it was possible to test their phylogeny for a linear temporal signal by the TempEst method—None was found. They gave a ‘x-intercept’ (i.e., TMRCA, Time to Most Recent Common Ancestor) of 3318.16 CE (Common Era) with a correlation coefficient of −0.136, which is, of course, nonsense. Therefore, the B cluster was tested separately by TempEst and LSD as it is of 76 concats with known collection dates, though some of these are uncertain as only their GenBank submission dates, not their collection dates, are recorded in GenBank and research papers. For these, we used their submission dates minus one year. The TempEst and LSD analyses both showed it to have a temporal signal. The B cluster seems to have evolved coherently in that it has an even distribution of nodes and branch lengths and includes both Peruvian and non-Peruvian isolates. They gave an intercept of 1593 CE with a correlation coefficient of 0.178 (*p* = 0.125). The TMRCA of the B cluster was also estimated by LSD and found to be 1451 CE (evolutionary rate: 0.68 × 109 CE–1855 CE; 1.1 × 10^−4^ s/s/year). However, when all non-synonymously changing codons were removed from the B cluster concats leaving only sequences of synonymously changing sequences (3822 nts long), they gave a nonsensical TMRCA of 3822 CE in a TempEst analysis.

The mean of the patristic distances connected through the root of the overall PVX ML phylogeny (Figure 2) is 15.86 times greater than the mean of those connected through the root of the B cluster (1.510 s/s ± 0.045 and 0.095 s/s ± 0.015, respectively), and this enables the TMRCA of PVX to be extrapolated from the TMRCA of the B cluster.

### 3.7. Origin of PVX

**Figure 7:**
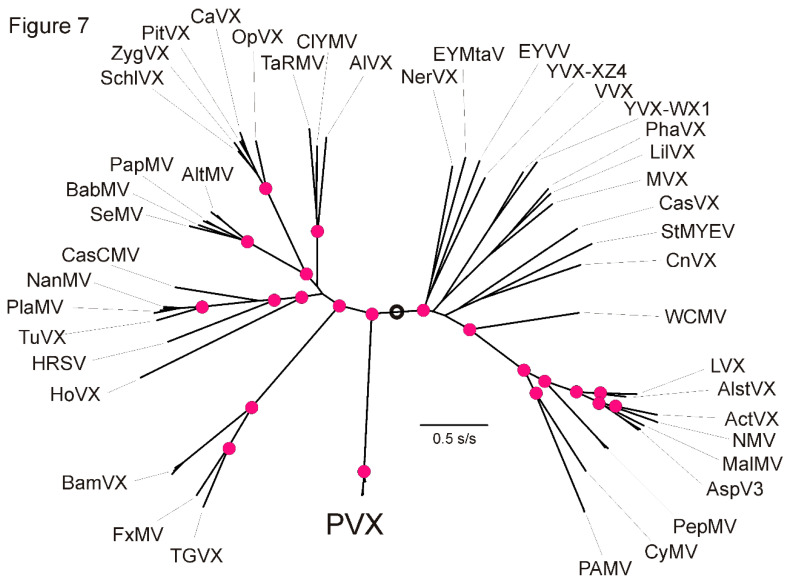
A maximum likelihood phylogeny of 44 potexviruses calculated from the concatenated nucleotide sequences of their replicase and coat protein genes. Midpoint of the phylogeny is circled. Acronyms and Accession Codes: ActVX, Actinidia virus X (KR872420); AlstVX, Alstroemeria virus X (NC_007408); AltMV, Alternanthera mosaic virus (GQ179647, LC107515, NC_007731); AlVX, Allium virus X (FJ670570); AspV3, Asparagus virus 3 (AB304848, KJ544560); BabMV, Babaco mosaic virus (MF978248); BamVX, Bamboo mosaic virus (AB636266, KU936346, KX648527, NC_001642); CasCMV, Cassava common mosaic virus (MN428639); CasVX, Cassava virus X (KY288487); CaVX, Cactus virus X (AF308158. JF937699); ClYMV, Clover yellow mosaic virus (D29630); CnVX, Cnidium virus X (LC460456); CyMV, Cymbidium mosaic virus (EF125180); EYMtaV, Euonymus yellow mottle associated virus (MK572000); EYVV, Euonymus yellow vein virus (NC_035190); FxMV, Foxtail mosaic virus (AY121833, MF573299, NC_001483); HoVX, Hosta virus X (NC_011544); HRSV, Hydrangea ringspot virus (NC_006943); LilVX, Lily virus X (NC_007192); LVX, Lettuce virus X (AM745758, NC_010832); MalMV(Chenopodium mosaic virus), NC_008251); MVX, Mint virus X (NC_006948); NanMV, Nandina mosaic virus (AY800279); NerVX, Nerine virus X (NC_007679); NMV, Narcissus mosaic virus (KF752593, NC_001441); OpVX, Opuntia virus X (KY348771, NC_006060); PAMV, Potato aucuba mosaic virus (KY123701, MG356506, NC_003632); PapMV, Papaya mosaic virus (D13957, MN203140, MN203142); PepMV, Pepino mosaic virus (FJ212288, JN133846, MT018444); PhaVX, Phaius virus X (NC_010295); PitVX, Pitaya virus X (NC_024458); PlaMV, Plantago asiatica mosaic virus (AB360796, LC155795, LC422371, NC_003849); PVX, Potato virus X (EU021215, KM659859, MT264741, X55802, MT708134, MT708143, MK116552); SchlVX, Schlumbergera virus X (NC_011659); SeMV, Senna mosaic virus (NC_030746); StMYEV, Strawberry mild yellow edge virus (AJ577359, NC_003794); TaRMV, Tamus red mosaic virus (JN389521); TGVX, Turtle grass virus X (MH077559, NC_040644); TuVX, Tulip virus X (NC_004322); VVX, Vanilla virus X (NC_035205); WCMV, White clover mosaic virus (X06728, X16636, MN814316); YVX-WX1, Yam virus X (NC_025252); YVX-XZ4, Yam virus X (1) (KJ789134); ZygVX, Zygocactus virus X (NC_006059).

We checked whether the geographical origins of PVX might be indicated by its phylogenetic relationships with other potexviruses, as comparisons of this sort had shown that PVA [58], PVY [59] and wild potato mosaic virus [99] had all evolved from lineages of potyviruses that were originally American. Figure 7 shows a ML phylogeny of 44 potexviruses calculated from the concatenated nt sequences of their replicase and CP genes. It can be seen that PVX forms a basal lineage of the potexviruses on a very long branch. None of the potexviruses have obvious phylogeographic groupings, except perhaps those infecting cacti (CaVX, OpVX, PitVX, SchlVX and ZygVX), which are an iconic South American group of plants, but are collected as a hobby, so their apparent grouping may reflect recent activity of hobbyists rather than their geographical origin. 

## 4. Discussion

Our analysis has provided important new information about the influence humans have inadvertently exerted upon the dispersion, diversity and evolution of PVX both within the potatoes’ Andean domestication center and within the rest of the world. This information was obtained through applying a combination of HTS, recombination, phylogenetic, population genetic, dating and other analyses to study for the first time an extensive virome consisting of PVX isolates from both potato′s domestication center in the Andean region and the rest of the world. The findings revealed the fingerprints of humans as a vector driving the global changes in the PVX population. These fingerprints included the periods both before and after potato, and along with it, PVX was dispersed far away from its original crop domestication center, resulting in acceleration of these changes. This improved understanding our findings have provided will benefit researchers in future similar studies with other economically important viruses dispersed away from their domestication centers to other parts of the world with their principal crop hosts. It will also benefit plant breeders, seed producers and marketers alike, in addressing the threat posed by virus diseases originally emanating from crop domestication centers.

The phylogeny of the large population composed of 370 non-rec sequences both confirmed earlier phylogenetic analyses of the smaller selection of sequences available then [42,43,100] and added to it. Both the 370 concat and 488 CP sequences were placed in two major (I, II) and five minor (I-1, I-2, II-1, II-2, II-3) phylogroups. Of these, I-2 (number of sequences, *n* = 3) and II-3 (*n* = 127) were of Andean isolates only, II-1 (*n* = 8) was of European isolates only and I-1 (*n* = 351) and II-2 (*n* = 43) were of isolates from both the Andes and elsewhere. Around half of the I-1 sequences, but only 10% of the II-2 sequences, were non-Andean. Considering that one of the well-sampled phylogroups, II-3 (*n* = 127), was of Andean sequences only, whereas the other well-sampled phylogroups, I-1 (*n* = 351) and II-2 (*n* = 43), were from both the Andes and elsewhere, it is likely that PVX first infected potatoes in the Andes and was spread from there to other parts of the world. Genetic diversity estimates (π) revealed that Andean minor phylogroups I-2 and II-3 were the most genetically diverse, indicating that they are the oldest, and the Tajima’s D static test returned significant negative values for I-1 and II-2, but not for II-3, indicating that the first two, but not the third, arose by expansion of migrants from the Andean population. Furthermore, a combination of Tajima′s D, F_ST_ and Nm analyses of PVX gene populations indicated that PVX most likely spread first from the Andes to Europe and Middle East, and then independently from there to East Asia and the Indian subcontinent. However, applying cladistic reasoning to the distribution of Andean and non-Andean PVX isolates in large sub-clusters B1 and B2 suggested that the migration was complex because, although PVX mostly spread from the Andes to other parts of the world, it likely did so on several occasions, and there had also been some more-recent PVX ‘repatriations’ to the Andes.

The dated sequences in the concat alignment yielded no detectable temporal signal in a TempEst analysis. We therefore studied Cluster B in more detail as it has isolates from both the Andes and elsewhere and TempEst and LSD analyses showed it to have a temporal signal. Its dated sequence yielded a TMRCA of c. 1593 CE, although with statistical support of only *p* = 0.178. Nonetheless, this is an entirely plausible date for PVX-infected tubers to have been transported in early shipments of potato tubers from the Andes to Europe as part of the ‘Columbian Exchange’ of crops between Europe and the Americas after their discovery by Columbus. This suggests that PVX became established in Europe well before the potato late-blight (*Phytophora infestans*) epidemic of the mid-19th century. The near simultaneous divergences of four large clusters of isolates (A, B, C, D) in the PVX phylogeny occurred during the same period as the major divergences found in the phylogenies of PVA, PVS and PVY [58,59,60]. These divergences all occurred around the mid-19th century following the famine-causing epidemics of late blight (*Phytophthora infestans*) in European potato crops in 1845–1849 [101,102]. The earliest potatoes carried to Europe lacked genetic diversity so, when the blight epidemic struck, almost all existing potato cultivars were killed. This greatly stimulated the breeding of new cultivars using potato germplasm, much of it imported from Chile in South America [48,103]. The surge in potato breeding and trade would have stimulated virus spread, and produced the divergences in the PVS, PVY and PVA populations [58,60,93], like that found by us in the PVX population. Therefore, there are two possible TMRCAs for Cluster B: either the poorly supported 1593 CE or the hypothetical 1868 CE. These may be extrapolated using patristic distances within the ML phylogeny of PVX (Figure 2) to provide two estimates of PVX TMRCA using the ratio of the mean patristic distance of the branch tips (=leaves) connected through the midpoint root (1.510 ± 0.045 substitutions/site) and those connected through Cluster B′s basal node (0.095 ± 0.015 substitutions/site), a ratio of 15.86. Thus, the ‘poorly supported TMRCA’ of PVX is around 6900 years ago, whereas the ‘hypothetical TMRCA’ is around 2380 years ago. Both of these are within the period since potato was first domesticated in the Andean region around 9000 years ago [104,105,106]. However, both are clearly earlier than the TMRCAs of PVY and PVA (c. 150 CE), when potato production increased during the Tiahuanaco (=Tiwanaku) empire, which lasted from 110 to about 1000 CE [46,107,108]. However, TMRCAs merely indicate the coalescence date of the variants in existing populations, and so may indicate that the smaller potato population of pre-Tiahuanaco times was able to sustain a more diverse PVX population of PVX than of either PVY or PVA.

Of the 388 PVX genomes studied, only 4.9% were found to be recombinant, which is an unexpectedly small percentage as genomes from comparable populations of two economically important potyviruses, PVY and turnip mosaic virus [59,109], have ten times as many rec sequences. Also, there was clear evidence that the Peruvian PVX population has been geographically well ‘mixed’, presumably by local trade in seed potatoes. Possibly, cross-protection occurring in mixed infections between PVX strains may limit recombination. However, the frequent occurrence of isolate mixtures within individual samples (326 genomic sequences obtained from 269 samples) collected in the field (Table 1b, Figure 1) suggest that this is unlikely to be important. Nonetheless, it might be useful if past studies on cross-protection by different strains of PVX [3,110] could be reinterpreted in the phylogenetic and geographic contexts our analyses have provided. None of PVX′s CP genes were found to be recombinant.

In our study, biological strain group 2 isolates were in minor phylogroups II-1 and II-2, strain group 1 and 3 isolates in major phylogroup I and strain group 4 isolates within all three of these groupings. This fits the pattern found previously by Cox and Jones [42]. What is new here is that major phylogroup I′s minor phylogroups I-1 and I-2 both contained isolates previously assigned to strain groups, those in I-2 coming solely from Peru and in I-1 being non-Andean. Absence of any strain groups in minor phylogroup II-3 reflects the lack of biological studies with any of the entirely new Peruvian isolates it consists of. Future research on Andean PVX isolates should include providing more information about the biological strain groups they belong to, especially those in minor phylogroup II-3.

Comparing historical isolate sequences with recent sequences of the same virus from the same part of the world can reveal whether regional alterations in virus populations have occurred with the passage of time [111]. For example, when genome sequences obtained from PVY isolates first isolated from potato in the period 1938–1984 in Western Europe were compared with recent ones: (i) none of the former belonged to the PVY rec sequences that have largely displaced their non-rec parents since their first appearance in the 1980s, (ii) no other examples of potato isolates belonging to the minor phylogroup PVY^C1^ found readily in 1939–1943 appeared subsequently and (iii) minor phylogroup PVY^C2^ became rare after the 1980s. Thus, a major population shift away from PVY^C1^ occurred over the last 80 years and of PVY^C2^ over the last 30 years [111,112,113,114] (note: minor phylogroups PVY^C1^ and PVY^C2^ were recently renamed PVY^C^ and PVY^O3^ respectively, by Fuentes et al. [59]). The oldest PVX isolate in the phylogeny is B (MT708134) isolated from potato cv. Duke of York in 1940 [24,115], which fits into minor phylogroup II-1. This was formerly the type isolate of potato virus B before this virus was considered to be a strain of PVX [116,117]. It belongs to PVX strain group 2 which was widely studied in the early days of Western European potato virus research [6,24,115], but by the 1980s was difficult to find except in old potato cultivars such as King Edward and Epicure [28,118]. The PVX isolates in minor phylogroup II-1 are all old ones from Western Europe. Therefore, there has been a major population decline in the occurrence of isolates within this minor phylogroup. This decline occurred due to the very widespread occurrence of PVX resistance gene *Nb* in Western European potato cultivars bred since the 1940s [29,56,115]. Establishing whether a similar decline in the phylogroup I-2 population has occurred in the Andean region would require future research on Andean PVX isolates to establish to which biological strain groups they belong. 

Our analyses of local inter-Departmental spread of PVX within Peru reveals that the most significant PVX gene flow (genetic linkage) was: firstly, between the Andean mountain Departments of Cajamarca, Huanuco, Junin, Huancavelica and Puno, where potatoes are grown under summer rainfall, secondly between the coastal sea level Departments of Ica and Lima, where potatoes are grown under irrigation in winter, and the mountain Departments closest to them that supply their seed potatoes (Junin for Lima; Huancavelica for Ica) (Figure 4).

Overall, we find many features of the Andean and world PVX populations are completely congruent with the hypothesis that humankind has been the principal long-distance vector of PVX from its origin within the Andean potato population. However, our analyses of the placement of PVX within the potexviruses give no clues on the origin of PVX, nor of the populations, hosts or world regions that were involved in its survival indicated by the very long branch connecting PVX to the base of the potexvirus phylogeny (Figure 7).

Among the common potato viruses, PVX is not one of the most damaging to the potato crop. Nevertheless, it infects potato worldwide: damaging severe PVX strains sometimes occur and mild PVX strains cause extremely damaging disease in mixed virus infections with PVA and PVY [7,8,13,15,16]. Also, PVX infection of potato plants has been reported to enhance their resistance to potato late-blight disease [119]. Moreover, minor phylogroups I-2 and II-3 were entirely Andean and, although biological studies have been undertaken with isolates from I-2 [30,31], none have been done with II-3 isolates. Also, only one of the 18 rec PVX isolates we found was from outside the Andean region (from the USA). Therefore, biological studies seem advisable to establish whether PVX rec isolates, and the non-rec isolates making up minor phylogroups I-2 and II-3, might be a potential cause for concern for potato-growing countries outside the Andean region. Following the completion of such studies, the appropriate biosecurity authorities of non-Andean countries would be in a position to consider whether precautions to prevent their establishment are required.

Existing systems for large-scale routine detection of common potato viruses, such as PVX, PVS, PVY, PVA and potato leaf roll virus (PLRV; genus *Polerovirus*, family, *Luteoviridae*), include using multiplex reverse transcription polymerase chain reaction (RT-PCR) assays to detect them simultaneously and quantitative real-time RT-PCR to provide greater sensitivity [120,121]. Our sequencing study involving many PVX isolates from the potato crop′s Andean potato domestication center, along with our earlier sequencing studies with PVS, PVY and PVA isolates from this region [58,59,60], have greatly increased the sequence diversity now available for each of these four viruses. To ensure greater reliability of future multiplex RT-PCR detection procedures, we recommend the preparation of new primer sets able to detect the increased PVX, PVS, PVY and PVA sequence diversity revealed by our studies. HTS has proven unsuitable for use in large-scale routine virus detection because of its prohibitive cost and the variable genome structure of RNA viruses, which constitutes a serious barrier to designing diagnostic markers that detect diverse plant virus species [122]. Fortunately, targeted genome sequencing (TC-Seq), an amplicon sequencing strategy involving a multiplex PCR reaction that not only detects diverse virus sequence targets simultaneously, but also greatly reduces cost and workload, holds considerable promise for sensitive, large-scale routine plant virus testing in both biosecurity and healthy stock programs in the future [122]. 

## Figures and Tables

**Table 1:** Origins of potato virus X (PVX) isolates newly sequenced in this study. (**a**) Historical sequences collected in 1940 to 1983. (**b**) Summary of Peruvian sequences from samples collected in 2016–2018.

(a)								
Isolate	Source Species	Cultivar/Breeding Line	Accession Number	Where Collected/Obtained	Isolation Year	Strain Group (=Pathotype)	GenBank Code	References
E	*S. tuberosum* subsp. *andigena*	Renacimiento	N/A	Central- southern highlands- Perú	1973	N/A	MT708135	[30]
CP (=C)	*S. tuberosum* subsp. *andigena*	Renacimiento	N/A	Central- southern highlands-Perú	1973	2	MT708142	[30]
CP4	*S. tuberosum* subsp. *andigena*	(Renacimiento)**	N/A	(Central- southern highlands-Perú)	(1973)	4	MT708141	[36]
DP (=D)	*S. goniocalyx*	Runtush	0CH 02736	Jauja, Junin, Department, Perú	1973	1 and 3 (mixture)	MT708143	[30]
A	*S. tuberosum* subsp. *andigena*	Ccompis	PI 308884	Wisconsin, USA in tuber from Peru *	1970	1 and 3(mixture)	MT708136	[30]
HB	*S. tuberosum* subsp. *andigena*	Suta	N/A	Puna, Potosi Department, Bolivia	1975	4	MT708134	[31]
B	*S. tuberosum* subsp. *tuberosum*	Duke of York	N/A	Scotland	1940	2	MT708140	[24,36]
DX	*S. tuberosum* subsp. tuberosum	Desiree	N/A	Cambridgeshire, England	1980	3	No sequence	[35]
DX4	*S. tuberosum* subsp. *tuberosum*	(Desiree)	N/A	(Cambridgeshire, England)	(1980)	4	MT708139	[35]
EX	*S. tuberosum* subsp. *tuberosum*	Epicure	N/A	Cambridgeshire, England	1983	2	MT708138	[28,36]
EX4	*S. tuberosum* subsp. *tuberosum*	(Epicure)	N/A	(Cambridgeshire, England)	(1983)	4	MT708137	[36]
(**b**)								
**Isolate Prefix**	**Peruvian Department** **Collected From**	**Year of Isolation**	**Total Samples**	**Number of Isolates Sequenced**				
Apu	Apurimac	2019	3	3				
Cca	Cajamarca	2016	60	67				
Cus	Cusco	2016	10	13				
Hua	Huancavelica	2016–2018	15	17				
Hco	Huanuco	2016	37	44				
Ica	Ica	2017	26	33				
Jin	Junin	2016	77	93				
Lim	Lima	2017	29	37				
Pun	Puno	2018	12	19				
**TOTAL**			269	326				

N/A = Not available. * Isolated in 1970 from a sprouted tuber received when CF was in Wisconsin. ** Round brackets surrounding a cultivar name mean that this isolate was derived from the isolate immediately above it.

**Table 2:** Potato virus X recombinants and their parents.

Recombinant (Rec)			Major Parent				Minor Parent				Rec. Region		RDP4 Programs ^1^	Method
Accession (Acc.) Code	Isolate	Collection Site ^2^	Acc. Code	Cluster	Isolate	Collection Site ^2^	Acc. Code	Cluster	Isolate	Collection Site ^2^	start	end		CRS ^3^
MT752615	Cca004-2	5	MT752839	B	Jin125	229	MT752614	G	Cca004-1	4	2602	2812	7	0.739
MT752631	Cca043	21	MT752857	rec	Jin163	247	MT752799	K	Jin051	189	3530	3664	6	0.672
MT752689	Cus089-2	79	MT752857	rec	Jin163	247	MT752757	K	Ica016	147	3458	3562	4	0.667
MT752729	Hco027-1	119	MT752785	F	Ica099-1	175	MT752872	B	Jin174-3	262	5634	6387	7	0.719
MT752730	Hco027-2	120	MT752873	B	Jin175	263	MT752785	F	Ica099-1	175	5634	6387	7	0.719
MT752758	Ica017-1	148	MT752611	G	Apu008	1	MT752791	A	Jin035	181	5078	5237	5	0.693
MT752761	Ica027-1	151	MT752790	L	Jin032	180	MT752763	rec	Ica027-3	153	6117	6210	6	0.742
MT752762	Ica027-2	152	MT752787	A	Ica100	177	MT752790	L	Jin032	180	2918	3095	5	0.59
MT752763	Ica027-3	153	MT752762	rec	Ica027-2	152	MT752761	rec	Ica027-1	151	3513	3661	7	0.581
MT752783	Ica098-1	173	MT752792	G	Jin041	182	AB196001	B	Japan	-	1580	1649	5	0.697
MT752857	Jin163	247	MT752846	C	Jin170B	236	MT752799	K	Jin051	189	6258	6387	6	0.66
MT752869	Jin173	259	MT752824	C	Jin109-2	214	MT752825	I	Jin110	215	2346	2502	6	0.737
MT752877	Jin178	267	MT752826	S	Jin110-B	216	MT752825	I	Jin110	215	2346	2601	7	0.738
MT752896	Lim084	286	MT752804	F	Jin059	194	MT752829	I	Jin113	219	5014	5521	7	0.696
MT752919	Pun001-2	309	MT752921	C	Pun002-2	311	MT708136	S	Peru	-^4^	5914	6073	6	0.644
MT752933	Pun035-2	323	MT752934	S	Pun035-3	324	MT752774	G	Ica040A	164	1542	1652	6	0.672
HQ450387	USA	-	unknown	-	unknown	-	M95516	B	UK	-	1436	3731	6	0.573
M31541	Peru	-	X55802	F	Argentina	-	unknown	-	-		5387	5929	4	0.701

^1^ Number of recombination methods in RDP that recorded that the recombinant region was significantly anomalous statistically. ^2^ Collection sites numbered in Figure 2. ^3^ CRS = Consensus Recombinant Score calculated by RDP4 program. ^4^ - = unknown.

**Table 3:** Genetic links (gene flow) between the potato virus X concat populations. (**a**) Genetic links between the concat populations of different continents. (**b**) Genetic links (gene flow) between the coat protein (CP) gene populations of different continents.

**(a)**							
**Continent**	***n***		**F_ST_**			**Nm**	
		Asia	Europe	Andean South America	Asia	Europe	Andean South America
Africa	6	0.079	0.201	0.318	2.88	0.99	0.54
Asia	15		0.189	0.308		1.07	0.56
Europe	17			0.073			3.19
Andean South America	346						
(**b**)							
**Continent**	***n***			**F_ST_**			
		East Asia	West Eurasia	Indian Subcontinent	Andean Region		
East Asia	37		0.041	0.113	0.316		
West Eurasia	53			0.069	0.205		
Indian Subcontinent	66				0.320		
Andean Region	313						

*n* = number of concats in the population; F_ST_ (coefficient of genetic differentiation), and Nm (gene flow parameter), both of which measure the genetic link between two populations. *n* = number of CP genes in the population; F_ST_ (coefficient of genetic differentiation), which measures the genetic link between two populations. Populations of seven African CP genes and nine Australian CP genes omitted as these populations were too small.

**Table 4:** Genetic links (gene flow) between the potato virus X concat populations of eight different Peruvian Departments.

Department ^a^	*n*					F_ST_			
		Cca	Cus	Hco	Hua	Ica	Jin	Lim	Pun
Apu	3	0.024	−0.224	0.045	−0.237	−0.137	−0.062	−0.109	−0.017
Cca	65		0.191	−0.008	0.183	0.116	0.021	0.075	−0.005
Cus	12			0.198	−0.048	−0.002	0.086	0.032	0.134
Hco	42				0.192	0.108	0.016	0.068	−0.016
Hua	17					0.004	0.086	0.031	0.126
Ica	28						0.027	−0.009	0.049
Jin	90							0.009	−0.009
Lim	36								0.0.12
Pun	17								

^a^ Apu: Apurimac, Cca: Cajamarca, Cus: Cusco, Hco: Huanuco, Hua: Huancavelica, Ica: Ica, Jin: Junin, Lim: Lima, Pun: Puno. Negative F_ST_ values are invalid and indicate either inadequate numbers of samples (e.g., Apu) or more variation within than between the populations being compared.

## Data Availability

The data is contained within the article or supplementary material.

## Supplementary Materials

The following are available online at https://www.mdpi.com/article/10.3390/v13040644/s1. Figure S1: The ML phylogeny of the concats of potato virus X Cluster B isolates. The Accession Codes of Peruvian isolates are in green, those of isolates from non-South American countries are in red, Table S1: Details of the origins of new potato virus X isolates sequenced from Peru in this study, Table S2: Genetic diversity of potato virus X population based on the coding genome sequence and each gene separately, File S1: Spread sheet version of Table 1 including additional potato virus X isolate data. Column A, Database record number; B, Accession Code; C, Peruvian isolate number (see Figure 1); D, Phylogroup (see Figure 2 and SM File S2); E, Cluster (see Figure 2); F, Isolate name; G, Provenance (country or Peruvian Department from which the isolate was collected); H, Continent of provenance (grouping used for DNAsp6 analysis); I, rec, recombinant, n-rec, non-recombinant (cluster not given for rec sequences); J, Collection date, “?” indicates submission date if collection date unknown; K, Original host of isolate; L, Host cultivar, if known; M, CP sequences analysed, File S2: The Accession Codes of the potato virus X isolates in the different clusters shown in Figure 2.
