# Supplementary material for: The Phylogeography of Potato Virus X Shows the Fingerprints of Its Human Vector
Source: Viruses. 2021 Apr 9;13(4):644. doi: 10.3390/v13040644 (PMC8070401; doi:10.3390/v13040644)
Supplement: Supplementary file 1 [file viruses-13-00644-s001.pdf]

Supplementary data

Table S1. Details of the origins of new potato virus X isolates sequenced from Peru in this study

Table S2. Genetic diversity of potato virus X population based on the coding genome sequence and each gene separately.

Figure S1. The ML phylogeny of the concats of potato virus X Cluster B isolates. The Accession Codes of Peruvian isolates are in green, those of isolates from non-South American countries are in red.

File S1. Spreadsheet version of Table 1 including additional potato virus X isolate data. Column A, Database record number; B, Accession Code; C, Peruvian isolate number (see Figure 1); D, Phylogroup (see Figure 2); E, Cluster, as in Figure 2 and S File 2; F, Isolate name; G, Provenance (country or Peruvian Department from which the isolate was collected); H, Continent of provenance (grouping used for DNAsp6 analysis); I, rec, recombinant, n-rec, non-recombinant (cluster not given for rec sequences); J, Collection date, ? indicates submission date if collection date unknown; K, Host of isolate; L, Host cultivar, if known; M, CP sequences analysed.

File S2. The Accession Codes of the potato virus X isolates in the different clusters shown in Figure 2.

Table S1. Details of the origins of new potato virus X isolates sequenced from Peru in this study

Isolate	Source Species	Cultivar/ breeding line	CIP accession number	Where collected /obtained	Isolation year	Strain group (=pathotype)	GeneBank code	References
Apu008<br>Apu010A<br>Apu100A	*Solanum × chaucha*	Putis	N/A	Abancay, Apurimac Deparment, Peru	2018	N/A	MT752611<br>MT752612<br>MT752613	This study
Cca004-1<br>Cca004-2	{(tbr x adg) x tbr} x a mixture of a mixture of pollen of early clones	Amarilis	N/A	Chota, Cajamarca Department, Peru	2016	N/A	MT752614<br>MT752615	This study
Cca005<br>Cca006	*S. tuberosum* ssp. *tuberosum* x *S. tuberosum* ssp. *andigena*	Perricholi	N/A	Chota, Cajamarca Department, Peru	2016	N/A	MT752616<br>MT752617	This study
Cca008<br>Cca009	{(tbr x adg) x tbr} x a mixture of a mixture of pollen of early clones	Amarilis	N/A	Chota, Cajamarca Department, Peru	2016	N/A	MT752618<br>MT752619	This study
Cca010	*S. tuberosum* ssp. *tuberosum* x *S. tuberosum* ssp. *andigena*	Perricholi	N/A	Chota, Cajamarca Department, Peru	2016	N/A	MT752620	This study

Cca010B	{(tbr x adg) x tbr} x a mixture of a mixture of pollen of early clones	Amarilis	N/A	Chota, Cajamarca Department, Peru	2016	N/A	MT752621	This study
Cca012<br>Cca016<br>Cca018<br>Cca020<br>Cca020B	*S. phureja*	Chaucha	N/A	Cutervo, Cajamarca Department, Peru	2016	N/A	MT752622<br>MT752623<br>MT752624<br>MT752625<br>MT752626	This study
Cca022	{(tbr x adg) x tbr} x a mixture of a mixture of pollen of early clones	Amarilis	N/A	Cutervo, Cajamarca Department, Peru	2016	N/A	MT752627	This study
Cca035<br>Cca037	tbr x {(phu x phu) x tbr}	Molinera	N/A	Cutervo, Cajamarca Department, Peru	2016	N/A	MT752628<br>MT752629	This study
Cca041<br>Cca043<br>Cca046B<br>Cca047<br>Cca048<br>Cca049	{(tbr x adg) x tbr} x a mixture of a mixture of pollen of early clones	Amarilis	N/A	Cutervo, Cajamarca Department, Peru	2016	N/A	MT752630<br>MT752631<br>MT752632<br>MT752633<br>MT752634<br>MT752635	This study

Cca051<br>Cca052<br>Cca053<br>Cca055<br>Cca056<br>Cca058	*S. tuberosum* subsp. *andigena* x *tuberosum*	Yungay	N/A	Cutervo, Cajamarca Department, Peru	2016	N/A	MT752636<br>MT752637<br>MT752638<br>MT752639<br>MT752640<br>MT752641	This study
Cca067<br>Cca068	*S. tuberosum* ssp. *tuberosum* x *S. tuberosum* ssp. *andigena*	Canchan	N/A	Cutervo, Cajamarca Department, Peru	2016	N/A	MT752642<br>MT752643	This study
Cca069<br>Cca070<br>Cca070B<br>Cca070C	{(tbr x adg) x tbr} x a mixture of a mixture of pollen of early clones	Amarilis	N/A	Cutervo, Cajamarca Department, Peru	2016	N/A	MT752644<br>MT752645<br>MT752646<br>MT752647	This study
Cca071-1<br>Cca071-2<br>Cca072<br>Cca075-1<br>Cca075-2<br>Cca078<br>Cca080E	*S. phureja*	Chaucha	N/A	Cutervo, Cajamarca Department, Peru	2016	N/A	MT752648<br>MT752649<br>MT752650<br>MT752651<br>MT752652<br>MT752653<br>MT752654	This study
Cca083<br>Cca085	*S. tuberosum* subsp. *andigena* x *tuberosum*	Yungay	N/A	Chota, Cajamarca Department, Peru	2016	N/A	MT752655<br>MT752656	This study
Cca094	{(tbr x adg) x tbr} x a mixture of a	Amarilis	N/A	Chota, Cajamarca Department, Peru	2016	N/A	MT752657	This study

	mixture of pollen of early clones							
Cca096<br>Cca099<br>Cca100<br>Cca100B	*S. phureja*	Chaucha	N/A	Chota, Cajamarca Department, Peru	2016	N/A	MT752658<br>MT752659<br>MT752660<br>MT752661	This study
Cca102<br>Cca104-1<br>Cca104-2<br>Cca107<br>Cca110C<br>Cca111<br>Cca118<br>Cca119-1<br>Cca119-2<br>Cca120<br>Cca120B	*S. phureja*	Chaucha	N/A	Chota, Cajamarca Department, Peru	2016	N/A	MT752662<br>MT752663<br>MT752664<br>MT752665<br>MT752666<br>MT752667<br>MT752668<br>MT752669<br>MT752670<br>MT752671<br>MT752672	This study
Cca122-1<br>Cca122-2<br>Cca123<br>Cca125-1<br>Cca125-2<br>Cca126<br>Cca128<br>Cca130C	*S. tuberosum* subsp. *andigena* x *tuberosum*	Yungay	N/A	Chota, Cajamarca Department, Peru	2016	N/A	MT752673<br>MT752674<br>MT752675<br>MT752676<br>MT752677<br>MT752678<br>MT752679<br>MT752680	This study

Cus009<br>Cus010	*S. tuberosum* subsp. *andigena* x *tuberosum*	Yungay	N/A	Anta, Cusco Department, Peru	2016	N/A	MT752681<br>MT752682	This study
Cus016-1<br>Cus016-2	*S. tuberosum* ssp. *tuberosum* x *S. tuberosum* ssp. *andigena*	Cica	N/A	Anta, Cusco Department, Peru	2016	N/A	MT752683<br>MT752684	This study
Cus075<br>Cus086<br>Cus088<br>Cus089-1<br>Cus089-2<br>Cus089-3	*S. tuberosum* subsp. *andigena*	Ccompis	N/A	Cusco, Cusco Department, Peru	2016	N/A	MT752685<br>MT752686<br>MT752687<br>MT752688<br>MT752689<br>MT752690	This study
Cus122<br>Cus127<br>Cus130B	*S. tuberosum* subsp. *andigena* x *tuberosum*	Yungay	N/A	Calca, Cusco Department, Peru	2016	N/A	MT752691<br>MT752692<br>MT752693	This study
Hua001<br>Hua002	{(tbr x adg) x adg}	Andina	N/A	Tayacaja, Huancavelica Department, Peru	2016	N/A	MT752694<br>MT752695	This study
Hua012<br>Hua013<br>Hua016<br>Hua017<br>Hua020	*S. tuberosum* subsp. *andigena* x *tuberosum*	Yungay	N/A	Tayacaja, Huancavelica Department, Peru	2016	N/A	MT752696<br>MT752697<br>MT752698<br>MT752699<br>MT752700	This study

Hua021<br>Hua026	*Solanum × chaucha*	Huayro	N/A	Huancavelica, Huancavelica Department, Peru	2018	N/A	MT752701<br>MT752702	This study
Hua051<br>Hua055-1<br>Hua055-2<br>Hua057-1<br>Hua057-2<br>Hua059<br>Hua060<br>Hua060A	*S. tuberosum* subsp. *andigena* x *tuberosum*	Yungay	N/A	Angaraes, Huancavelica Department, Peru	2018	N/A	MT752703<br>MT752704<br>MT752705<br>MT752706<br>MT752707<br>MT752708<br>MT752709<br>MT752710	This study
Hco001<br>Hco002-1<br>Hco002-2<br>Hco004<br>Hco007	*S. tuberosum* subsp. *andigena* x *tuberosum*	Yungay	N/A	Ambo, Huanuco Department, Peru	2016	N/A	MT752711<br>MT752712<br>MT752713<br>MT752714<br>MT752716	This study
Hco005B	*S. stenotomum* subsp. *goniocalyx*	Tumbay	N/A	Ambo, Huanuco Department, Peru	2016	N/A	MT752715	This study
Hco012<br>Hco013<br>Hco015<br>Hco016<br>Hco019	*S. tuberosum* subsp. *andigena* x *tuberosum*	Yungay	N/A	Huanuco, Huanuco Department, Peru	2016	N/A	MT752717<br>MT752718<br>MT752719<br>MT752720<br>MT752721	This study
Hco021-1<br>Hco021-2<br>Hco022	*S. tuberosum* ssp. *tuberosum* x	Canchan	N/A	Huanuco, Huanuco Department, Peru	2016	N/A	MT752722<br>MT752723<br>MT752724	This study

Hco023	*S. tuberosum* ssp.						MT752725	
Hco025	*andigena*						MT752726	
Hco026-1							MT752727	
Hco026-2							MT752728	
Hco027-1							MT752729	
Hco027-2							MT752730	
Hco028							MT752731	
Hco029							MT752732	
Hco030B							MT752733	
Hco031	*S. tuberosum*	Yungay	N/A	Pachitea,	2016	N/A	MT752734	This study
Hco032	subsp. *andigena* x			Huanuco			MT752735	
Hco033	*tuberosum*			Department, Peru			MT752736	
Hco034							MT752737	
Hco035							MT752738	
Hco036							MT752739	
Hco038-1							MT752740	
Hco038-2							MT752741	
Hco039-1							MT752742	
Hco039-2							MT752743	
Hco054							MT752744	
Hco056							MT752745	
Hco057							MT752746	
Hco058							MT752747	
Hco059-1							MT752748	
Hco059-2							MT752749	

Hco062<br>Hco066<br>Hco067<br>Hco068<br>Hco070	*S. tuberosum* subsp. *andigena* x *tuberosum*	Yungay	N/A	Huanuco, Huanuco Department, Peru	2016	N/A	MT752750<br>MT752751<br>MT752752<br>MT752753<br>MT752754	This study
Ica011<br>Ica012<br>Ica016<br>Ica017-1<br>Ica017-2	[{(tbr x S. raphanifolium) x tbr} x adg] x tbr	Unica	N/A	Chincha, Ica Department, Peru	2017	N/A	MT752755<br>MT752756<br>MT752757<br>MT752758<br>MT752759	This study
Ica024<br>Ica027-1<br>Ica027-2<br>Ica027-3<br>Ica028<br>Ica029<br>Ica030A<br>Ica031-1<br>Ica031-2<br>Ica035<br>Ica036<br>Ica037<br>Ica039<br>Ica040<br>Ica040A<br>Ica045	*S. tuberosum* ssp. *tuberosum* x *S. tuberosum* ssp. *andigena*	Canchan	N/A	Pisco, Ica Department, Peru	2017	N/A	MT752760<br>MT752761<br>MT752762<br>MT752763<br>MT752764<br>MT752765<br>MT752766<br>MT752767<br>MT752768<br>MT752769<br>MT752770<br>MT752771<br>MT752772<br>MT752773<br>MT752774<br>MT752775	This study

Ica049							MT752776	
Ica052<br>Ica055	[{(tbr x S. raphanifolium) x tbr} x adg] x tbr	Unica	N/A	Ica, Ica Department, Peru	2017	N/A	MT752777<br>MT752778	This study
Ica091<br>Ica095<br>Ica096-1<br>Ica096-2<br>Ica098-1<br>Ica098-2<br>Ica099-1<br>Ica099-2<br>Ica100	*S. tuberosum* subsp. *andigena* x *tuberosum*	Yungay	N/A	Ica, Ica Department, Peru	2017	N/A	MT752779<br>MT752780<br>MT752781<br>MT752782<br>MT752783<br>MT752784<br>MT752785<br>MT752786<br>MT752787	This study
Jin014<br>Jin017<br>Jin032<br>Jin035	*S. tuberosum* subsp. *andigena* x *tuberosum*	Yungay	N/A	Tarma, Junin Department, Peru	2016	N/A	MT752788<br>MT752789<br>MT752790<br>MT752791	This study
Jin041<br>Jin042-1<br>Jin042-2<br>Jin043<br>Jin045<br>Jin046<br>Jin048<br>Jin051	[{(tbr x S. raphanifolium) x tbr} x adg] x tbr	Unica	N/A	Tarma, Junin Department, Peru	2016	N/A	MT752792<br>MT752793<br>MT752794<br>MT752795<br>MT752796<br>MT752797<br>MT752798<br>MT752799	This study

Jin054<br>Jin056<br>Jin057-1<br>Jin057-2<br>Jin059							MT752800<br>MT752801<br>MT752802<br>MT752803<br>MT752804	
Jin061	*S. tuberosum* subsp. *andigena* x *tuberosum*	Yungay	N/A	Tarma, Junin Department, Peru	2016	N/A	MT752805	This study
Jin075	[{(tbr x S. raphanifolium) x tbr} x adg] x tbr	Unica	N/A	Tarma, Junin Department, Peru	2016	N/A	MT752806	This study
Jin089<br>Jin090	*S. tuberosum* ssp. *tuberosum* x *S. tuberosum* ssp. *andigena*	Perricholi	N/A	Concepcion, Junin Department, Peru	2016	N/A	MT752807<br>MT752808	This study
Jin091<br>Jin095<br>Jin099	{(tbr x adg) x adg}	Andina	N/A	Huancayo, Junin Department, Peru	2016	N/A	MT752809<br>MT752810<br>MT752811	This study
Jin101-1<br>Jin101-2<br>Jin102<br>Jin103<br>Jin104<br>Jin105-1<br>Jin105-2<br>Jin106-1	*S. tuberosum* subsp. *andigena* x *tuberosum*	Yungay	N/A	Concepcion, Junin Department, Peru	2016	N/A	MT752812<br>MT752813<br>MT752814<br>MT752815<br>MT752816<br>MT752817<br>MT752818<br>MT752819	This study

Jin106-2<br>Jin107<br>Jin108<br>Jin109-1<br>Jin109-2<br>Jin110<br>Jin110B<br>Jin111<br>Jin112<br>Jin113<br>Jin114<br>Jin116<br>Jin118-1<br>Jin118-2<br>Jin119<br>Jin120<br>Jin120B							MT752820<br>MT752821<br>MT752822<br>MT752823<br>MT752824<br>MT752825<br>MT752826<br>MT752827<br>MT752828<br>MT752829<br>MT752830<br>MT752831<br>MT752832<br>MT752833<br>MT752834<br>MT752835<br>MT752836	
Jin122<br>Jin124<br>Jin125	*S. tuberosum* ssp. *tuberosum* x *S. tuberosum* ssp. *andigena*	Perricholi	N/A	Huancayo, Junin Department, Peru	2016	N/A	MT752837<br>MT752838<br>MT752839	This study
Jin126<br>Jin128<br>Jin129<br>Jin130<br>Jin130B	*S. tuberosum* ssp. *tuberosum* x *S. tuberosum* ssp. *andigena*	Canchan	N/A	Huancayo, Junin Department, Peru	2016	N/A	MT752840<br>MT752841<br>MT752842<br>MT752843<br>MT752844	This study

Jin131<br>Jin132<br>Jin133<br>Jin134-1<br>Jin134-2<br>Jin137<br>Jin139-1<br>Jin139-2<br>Jin140<br>Jin161-1<br>Jin161-2<br>Jin162<br>Jin163<br>Jin164<br>Jin166<br>Jin168<br>Jin169<br>Jin170-1<br>Jin170-2<br>Jin170B	*S. tuberosum* subsp. *andigena* x *tuberosum*	Yungay	N/A	Huancayo, Junin Department, Peru	2016	N/A	MT752845<br>MT752846<br>MT752847<br>MT752848<br>MT752849<br>MT752850<br>MT752851<br>MT752852<br>MT752853<br>MT752854<br>MT752855<br>MT752856<br>MT752857<br>MT752858<br>MT752859<br>MT752860<br>MT752861<br>MT752862<br>MT752863<br>MT752864	This study
Jin171-1<br>Jin171-2<br>Jin172-1<br>Jin172-2<br>Jin173<br>Jin174-1<br>Jin174-2	*S. tuberosum* subsp. *andigena* x *tuberosum*	Yungay	N/A	Chupaca, Junin Department, Peru	2016	N/A	MT752865<br>MT752866<br>MT752867<br>MT752868<br>MT752869<br>MT752870<br>MT752871	This study

Jin174-3<br>Jin175<br>Jin176<br>Jin177-1<br>Jin177-2<br>Jin178<br>Jin179<br>Jin180<br>Jin180B							MT752872<br>MT752873<br>MT752874<br>MT752875<br>MT752876<br>MT752877<br>MT752878<br>MT752879<br>MT752880	
Lim001<br>Lim002<br>Lim003<br>Lim027	[{(tbr x S. raphanifolium) x tbr} x adg] x tbr	Unica	N/A	Barranca, Lima Department, Peru	2017	N/A	MT752881<br>MT752882<br>MT752883<br>MT752884	This study
Lim033	*S. tuberosum* ssp. *tuberosum* x *S. tuberosum* ssp. *andigena*	Canchan	N/A	Barranca, Lima Department, Peru	2017	N/A	MT752885	This study
Lim041<br>Lim042<br>Lim043<br>Lim045<br>Lim046<br>Lim048<br>Lim050-1<br>Lim050-2<br>Lim050A	[{(tbr x S. raphanifolium) x tbr} x adg] x tbr	Unica	N/A	Barranca, Lima Department, Peru	2017	N/A	MT752886<br>MT752887<br>MT752888<br>MT752889<br>MT752890<br>MT752891<br>MT752892<br>MT752893<br>MT752894	This study

Lim071<br>Lim084<br>Lim086<br>Lim088<br>Lim089<br>Lim090	[{(tbr x S. raphanifolium) x tbr} x adg] x tbr	Unica	N/A	Huaral, Lima Department, Peru	2017	N/A	MT752895<br>MT752896<br>MT752897<br>MT752898<br>MT752899<br>MT752900	This study
Lim091-1<br>Lim091-2<br>Lim092<br>Lim093<br>Lim094<br>Lim095<br>Lim096-1<br>Lim096-2<br>Lim096-3<br>Lim097-1<br>Lim097-2<br>Lim097-3<br>Lim098<br>Lim099-1<br>Lim099-2<br>Lim100-1<br>Lim100-2	[{(tbr x S. raphanifolium) x tbr} x adg] x tbr	Unica	N/A	Cañete, Lima Department, Peru	2017	N/A	MT752901<br>MT752902<br>MT752903<br>MT752904<br>MT752905<br>MT752906<br>MT752907<br>MT752908<br>MT752909<br>MT752910<br>MT752911<br>MT752912<br>MT752913<br>MT752914<br>MT752915<br>MT752916<br>MT752917	This study
Pun001-1<br>Pun001-2<br>Pun002-1	*S. tuberosum* subsp. *andigena*	Huaycha	N/A	Yunguyo, Puno Department, Peru	2018	N/A	MT752918<br>MT752919<br>MT752920	This study

Pun002-2<br>Pun003-1<br>Pun003-2<br>Pun004							MT752921<br>MT752922<br>MT752923<br>MT752924	
Pun015<br>Pun019	*S. tuberosum* subsp. *andigena*	Ccompis	N/A	Chucuito, Puno Department, Peru	2018	N/A	MT752925<br>MT752926	This study
Pun031<br>Pun032-1<br>Pun032-2<br>Pun033-1<br>Pun033-2<br>Pun035-1<br>Pun035-2<br>Pun035-3<br>Pun036<br>Pun040	Unknown	Improved potato variety	N/A	Puno, Puno Department, Peru	2018	N/A	MT752927<br>MT752928<br>MT752929<br>MT752930<br>MT752931<br>MT752932<br>MT752933<br>MT752934<br>MT752935<br>MT752936	This study

Table S2. Genetic diversity of PVX population based on the coding genome sequence and each gene separately.

- Concat sequence (6357 nts)

Minor Phylogroups	N	π	SS	NS	d_S	d_N	d_N/d_S	Tajima's D[a]
I-1	203	0.031	1448.79	4812.21	0.118	0.005	0.040	-2.033*
I-2	3	0.153	1452.28	4859.72	0.576	0.027	0.047	ND
II-1	3	0.013	1482.72	4865.28	0.048	0.003	0.056	ND
II-2	37	0.037	1460.15	4824.85	0.1421	0.005	0.038	-1.841*
II-3	123	0.048	1470.82	4841.18	0.188	0.005	0.027	-1.358ns
All	369	0.171	1444.76	4789.24	0.474	0.035	0.081	0.478ns

- RdRP (4368 nts)

Minor Phylogroups	N	π	SS	NS	d_S	d_N	d_N/d_S	Tajima's D[a]
I-1	203	0.034	968.26	3348.74	0.135	0.005	0.035	-1.957*
I-2	3	0.162	976.39	3391.61	0.633	0.026	0.041	ND

II-1	3	0.014	990.22	3377.78	0.055	0.002	0.029	ND
II-2	37	0.038	983.76	3384.24	0.149	0.004	0.030	-1.819*
II-3	123	0.051	984.70	3383.30	0.210	0.005	0.024	-1.307ns
All	369	0.144	970.14	3346.86	0.510	0.038	0.074	0.565ns

- TGB1 (678 nts)

Minor Phylogroups	N	π	SS	NS	d_S	d_N	d_N/d_S	Tajima's D[a]
I-1	203	0.027	167.86	510.14	0.092	0.006	0.062	-2.137**
I-2	3	0.157	168.72	509.28	0.523	0.036	0.069	ND
II-1	3	0.014	172.17	505.83	0.042	0.004	0.093	ND
II-2	37	0.036	172.68	505.32	0.126	0.005	0.042	-1.893*
II-3	123	0.045	172.57	505.43	0.164	0.004	0.023	-1.344ns
All	369	0.143	169.95	508.05	0.457	0.038	0.083	0.431ns

- TGB2 (345 nts)

Minor Phylogroups	N	π	SS	NS	d_S	d_N	d_N/d_S	Tajima's D[a]
I-1	203	0.026	87.42	257.58	0.093	0.004	0.043	-1.909*
I-2	3	0.097	83.67	261.33	0.305	0.030	0.098	ND
II-1	3	0.010	85.22	259.78	0.031	0.003	0.082	ND
II-2	37	0.035	86.57	258.43	0.107	0.012	0.112	-1.747ns
II-3	123	0.037	88.28	256.72	0.124	0.007	0.060	-1.349ns
All	369	0.111	87.57	257.43	0.332	0.035	0.106	0.317ns

- TG3 (213 nts)

Minor Phylogroups	N	π	SS	NS	d_S	d_N	d_N/d_S	Tajima's D[a]
I-1	203	0.016	52.51	157.49	0.040	0.008	0.206	-2.458**
I-2	3	0.127	52.17	157.83	0.323	0.062	0.193	ND
II-1	3	0.009	51.89	161.11	0.026	0.004	0.161	ND

II-2	37	0.042	52.56	157.44	0.125	0.014	0.116	-1.707ns
II-3	123	0.033	51.26	158.74	0.089	0.014	0.156	-1.643ns
All	369	0.144	52.08	157.92	0.299	0.093	0.312	0.262ns

- CP (753 nts)

Minor Phylogroups	N	π	SS	NS	d_S	d_N	d_N/d_S	Tajima's D[a]
I-1	203	0.024	172.75	538.25	0.089	0.003	0.036	-2.232**
I-2	3	0.132	171.33	539.67	0.508	0.012	0.024	ND
II-1	3	0.012	183.22	560.78	0.025	0.007	0.280	ND
II-2	37	0.037	164.58	519.42	0.137	0.006	0.041	-1.908*
II-3	123	0.038	174.02	536.98	0.148	0.002	0.016	-1.568ns
All	369	0.121	165.01	518.99	0.409	0.029	0.070	0.077ns

[a]ND: Not determined; ns: Not significant, * 0.1 probability; ** 0.01 probability; N: Number of sequences, π: Nucleotide diversity, SS: Number of synonymous sites, NS: Number of non-synonymous sites, d_N: Non-synonymous nucleotide diversity, d_S: Synonymous nucleotide diversity and d_N/d_S: the ratio of non-synonymous nucleotide diversity to synonymous nucleotide diversity.

Figure S1. The ML phylogeny of potato virus X Cluster B isolates. The Accession Codes of Peruvian isolates are in green, those of isolates from non-South American countries are in red.

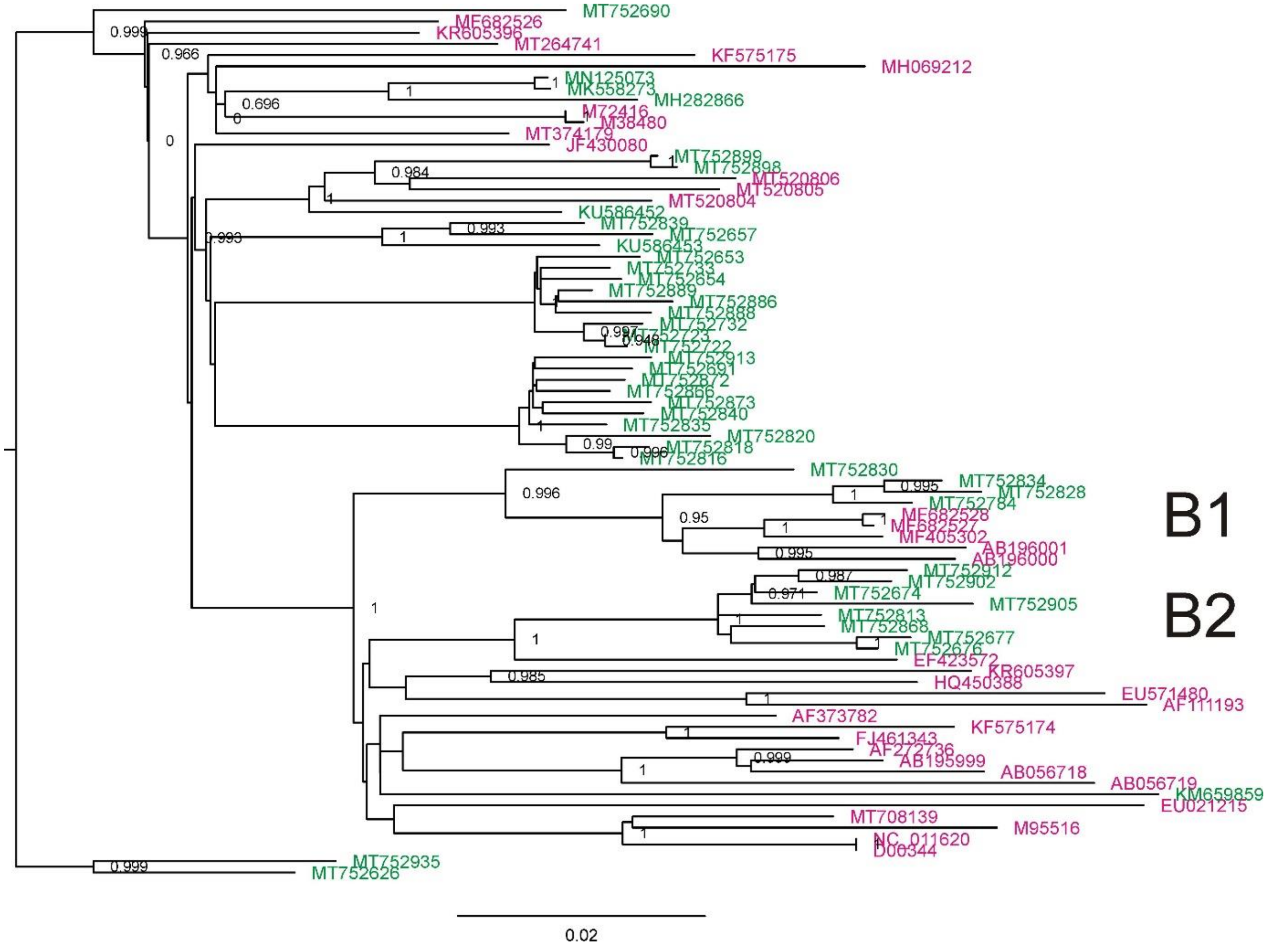

File S1 Spreadsheet version of Table 1 including additional potato virus X isolate data. Column A, Database record number; B, Accession Code; C, Peruvian isolate number (see Fig 1); D, Phylogroup (see Fig 2); E, Cluster, as in Fig 2 and SD File 2; F, Isolate name; G, Provenance (country or Peruvian Department from which the isolate was collected); H, Continent of provenance (grouping used for DNAsp6 analysis); I, rec, recombinant, n-rec, non-recombinant (cluster not given for rec sequences); J, Collection date, ? indicates submission date if collection date unknown; K, Host of isolate; L, Host cultivar, if known; M, CP sequences analysed.

A	B	C	D	E	F	G	H	I	J	K	L	M
dB record	Acc Code	Peru Isol No	Minor phylogroup	luster	Isol name	Provenance	continent	Rec-n-rec	Collection Date	Source Host	Cultivar	CP seqs
1	AB056718		I-1	B	os	Japan	Asia	n-rec	?2002	S.t.t		AB056718
2	AB056719		I-1	B	bs	Japan	Asia	n-rec	?2005	S.t.t		AB056719
3	AB195999		I-1	B	BH	Japan	Asia	n-rec	?2005	S.t.t		AB195999
4	AB196000		I-1	B	OG	Japan	Asia	n-rec	?2005	S.t.t		AB196000
5	AB196001		I-1	B	TO	Japan	Asia	n-rec	?2005	S.t.t		AB196001
6			I-1				Asia					AB451181
7	AF111193		I-1	B	ROTH1	UK	Eurasia	n-rec	?1998	S.t.t		AF111193
8	AF172259		II-2	F	CP4	UK	Eurasia	n-rec	?1999	S.t.t.		AF172259
9			I-1				North Am					AF202462
10			I-1				Asia					AF260640
11			I-1				Asia					AF260641
12	AF272736		I-1	B	Taiwan	Taiwan	Asia	n-rec	?2005	S.t.t.		AF272736
13	AF373782		I-1	B	KR	Korea	Asia	n-rec	?2001	S.t.t.		AF373782
14			I-1				Asia					AF485891
15			I-1				Eurasia					AF493949
16			I-1				Asia					AF528555
17			I-1				Asia					AF534912

18			I-1				Eurasia					AJ505748
19			I-1				Africa					
20	D00344		I-1	B	X3	Netherlands	Eurasia	n-rec	?1988	N.tab		
21			I-1				Asia					DQ315386
22			I-1				Asia					EF063709
23	EF423572		I-1	B	FX21	China	Asia	n-rec	?2008	S.t.t		EF423572
24	EU021215		I-1	B	Tula	Russia	Eurasia	n-rec	?2007			EU021215
25			I-1				Asia					EU031437
26			I-1				Asia					EU095494
27	EU571480		I-1	B	Tai'an	China	Asia	n-rec	1985			EU571480
28	FJ461343		I-1	B	Iran	Iran	Eurasia	n-rec	2004	Pisum sat		FJ461343
29			I-1				IPB					FJ643623
30			I-1				Eurasia					GQ496608
31			I-1				Eurasia					GU144346
32			I-1				Eurasia					GU144347
33			I-1				Eurasia					GU144348
34			I-1				Eurasia					GU144349
35			I-1				Eurasia					GU144350
36			I-1				Eurasia					GU144351
37			I-1				Eurasia					GU144352
38			I-1				Eurasia					GU144353
39			I-1				Eurasia					GU144354
40			I-1				Eurasia					GU144360
41			I-1				Eurasia					GU144361
42			I-1				Eurasia					GU144362
43			I-1				Eurasia					GU144363

44			I-1				Eurasia					GU256064
45			I-1				Asia					GU373815
46			I-1				Australia					GU384726
47			II-2				Australia					GU384729
48			I-1				Australia					GU384730
49			I-1				Australia					GU384731
50			I-1				Australia					GU384732
51			I-1				Australia					GU384733
52			I-1				Australia					GU384734
53			I-1				Australia					GU384735
54			I-1				Australia					GU384736
55			II-1				Eurasia					GU384737
56			II-1				Eurasia					GU384738
57			I-1				Eurasia					HG518656
58			I-1				Asia					HM036193
59			I-1				Asia					HM036194
60			I-1				Asia					HM036195
61			I-1				Asia					HM036196
62			I-1				Asia					HM036197
63			I-1				Asia					HM036198
64			I-1				Asia					HM036199
65			I-1				South Am					HQ433255
66	HQ450387		I-1		PVX2.7	USA	North Am	rec	2006	A.thaliana		HQ450387
67	HQ450388		I-1	B	PVX2.7b	USA	North Am	n-rec	2006	N.glut		
68	JF430080		I-1	B	Pt Del-9	India	IPB	n-rec	2009	S.t.t		

69			I-1				IPB					JF430080
70			I-1				IPB					JX273242
71			I-1				Eurasia					JX905355
72			I-1				IPB					KC757709
73			I-1				Eurasia					KC875236
74			I-1				Asia					KF225469
75			I-1				Asia					KF280643
76			I-1				Eurasia					KF537623
77			I-1				Eurasia					KF568897
78			I-1				Eurasia					KF568898
79			I-1				Eurasia					KF568899
80			I-1				Eurasia					KF568900
81			I-1				Eurasia					KF568901
82			I-1				Eurasia					KF568903
83			I-1				Eurasia					KF568904
84			I-1				Eurasia					KF568905
85			I-1				Eurasia					KF568906
86	KF575174		I-1	B	KER.ZA.1	Iran	Eurasia	n-rec	2011	S.t.t		KF575174
87	KF575175		I-1	B	KER.LA.2	Iran	Eurasia	n-rec	2010	S.t.t		KF575175
88	KJ534601		II-3	G	SA-CIP	Peru	South Am	n-rec	2011	S.t.t.Dat		KJ534601
89	KJ534602		II-2	F	GAFF224	Peru	South Am	n-rec	2011	S.t.t.		KJ534602
90	KJ534603		II-3	G	GAF225	Peru	South Am	n-rec	2011	S.t.t.		KJ534603
91	KJ534604		I-1	C	GAFF225_EuA	Peru	South Am	n-rec	2011	S.t.t.		KJ534604
92	KJ534605		II-2	F	GAFF226	Peru	South Am	n-rec	2011	S.t.t.		KJ534605
93			I-1				Eurasia					KJ631111
94	KM659859		I-1	B	Gooseberry	Colombia	South Am	n-rec	2014	P.peruv		KM659859

95			I-1				IPB					KR605330
96			I-1				IPB					KR605331
97			I-1				IPB					KR605332
98			I-1				IPB					KR605334
99			I-1				IPB					KR605336
100			I-1				IPB					KR605337
101			I-1				IPB					KR605338
102			I-1				IPB					KR605339
103			I-1				IPB					KR605340
104			I-1				IPB					KR605341
105			I-1				IPB					KR605342
106			I-1				IPB					KR605343
107			I-1				IPB					KR605344
108			I-1				IPB					KR605345
109			I-1				IPB					KR605346
110			I-1				IPB					KR605347
111			I-1				IPB					KR605348
112			I-1				IPB					KR605349
113			I-1				IPB					KR605351
114			I-1				IPB					KR605352
115			I-1				IPB					KR605353
116			I-1				IPB					KR605354
117			I-1				IPB					KR605355
118			I-1				IPB					KR605356
119			I-1				IPB					KR605358
120			I-1				IPB					KR605359

121			I-1				IPB					KR605360
122			I-1				IPB					KR605361
123			I-1				IPB					KR605362
124			I-1				IPB					KR605363
125			I-1				IPB					KR605364
126			I-1				IPB					KR605365
127			I-1				IPB					KR605366
128			I-1				IPB					KR605367
129			I-1				IPB					KR605368
130			I-1				IPB					KR605369
131			I-1				IPB					KR605370
132			I-1				IPB					KR605371
133			I-1				IPB					KR605372
134			I-1				IPB					KR605373
135			I-1				IPB					KR605374
136			I-1				IPB					KR605375
137			I-1				IPB					KR605376
138			I-1				IPB					KR605377
139			I-1				IPB					KR605378
140			I-1				IPB					KR605379
141			I-1				IPB					KR605380
142			I-1				IPB					KR605381
143			I-1				IPB					KR605382
144			I-1				IPB					KR605383
145			I-1				IPB					KR605384
146			I-1				IPB					KR605385

147			I-1				IPB					KR605386
148			I-1				IPB					KR605387
149			I-1				IPB					KR605388
150			I-1				IPB					KR605390
151			I-1				IPB					KR605391
152			I-1				IPB					KR605392
153			I-1				IPB					KR605394
154	KR605396		I-1	B	JAL-2	India	IPB	n-rec	2013	S.t.t		KR605396
155	KR605397		I-1	B	KUF-5	India	IPB	n-rec	2012	S.t.t		KR605397
156			I-1				South Am					KT923123
157	KU586452		I-1	B	GAF318-4.1	Peru	South Am	n-rec	?2016	S.t.t		KU586452
158	KU586453		I-1	B	GAF318-16.2	Peru	South Am	n-rec	?2016			KU586453
159			I-1				Eurasia					KY490355
160	M31541		II-2		CP	Peru-Neth	South Am	rec	?1998			
161	M38480		I-1	B		Russia	Eurasia	n-rec	?1988			
162			I-1				Eurasia					M38655
163	M72416		I-1	B		Russia	Eurasia	n-rec	?1987			M72416
164	M95516		I-1	B		UK	Eurasia	n-rec	1992	S.t.t		M95516
165	MF405302		I-1	B	CH	Switz	Eurasia	n-rec	2013	S.t.t		MF405302
166			I-1				IPB					MF589763
167	MF682526		I-1	B	Tn145	Tunisia	Africa	n-rec	2013	S.t.t		MF682526
168	MF682527		I-1	B	Tn147	Tunisia	Africa	n-rec	2013	S.t.t		MF682527
169	MF682528		I-1	B	Tn148	Tunisia	Africa	n-rec	2013	S.t.t		
170	MH069212		I-1	B	PVX2	Canada	North Am	n-rec	2005	S.t.t		MH069212
171	MH282866		I-1	B	Tomato-Antio	Colombia	South Am	n-rec	2018	S.lyco		MH282866
172			I-1				Asia					MH706740

173	MK116552		II-3	S	MyySE	Colombia	South Am	n-rec	2018	S. phureja		MK116552
174			I-1				Asia					MK387314
175	MK558273		I-1	B	May4A	Colombia	South Am	n-rec	2018	S.t.t		MK558273
176			I-1				Asia					MK587458
177			I-1				Asia					MK587459
178			I-1				Asia					MK672914
179	MN125073		I-1	B	PVXquit_M6	Colombia	South Am	n-rec	2019	S.quitoense		MN125073
180			I-1				North Am					MN688138
181			I-1				North Am					MN688139
182			I-1				Africa					MN689496
183			I-1				Eurasia					MN702769
184	MT264741		I-1	B	PO59	Ireland	Eurasia	n-rec	2017	S.t.t		MT264741
185	MT374179		I-1	B	DSMZPV-0014	Germany	Eurasia	n-rec	?2020	S.t.t		MT374179
186	MT520804		I-1	B	JEO11-25	Burundi	Africa	n-rec	2016	tuberosum	Ndinamagara (Cruza 148)	MT520804
187	MT520805		I-1	B	JEO11-30	Burundi	Africa	n-rec	2016	tuberosum	Ndinamagara (Cruza 148)	MT520805
188	MT520806		I-1	B	JEO11-14	Burundi	Africa	n-rec	2016	tuberosum	Ndinamagara (Cruza 148)	MT520806
189	MT708134		II-2	S	HB	Bolivia	South Am	n-rec	1975	Andigena	Suta	MT708134
190	MT708135		I-2	S	E	Peru	South Am	n-rec	1973	Andigena	Renacimiento	MT708135
191	MT708136		I-2	S	E	Peru-USA	South Am	n-rec	1970	Andigena	Ccompis	MT708136
192	MT708137		II-1	E	EX4	UK	Eurasia	n-rec	1983	tuberosum	(Epicure)	
193	MT708138		II-1	E	EX	UK	Eurasia	n-rec	1983	tuberosum	Epicure	
194	MT708139		I-1	B	DX4	U.K.	Eurasia	n-rec	1980	tuberosum	(Desiree)	MT708139
195	MT708140		II-1	E	B	Scotland	Eurasia	n-rec	1940	tuberosum	Duke of York	

196	MT708141		II-2	F	CP4	Peru	South Am	n-rec	1973	Andigena	(Renacimiento)	MT708141
197	MT708142		II-2	F	CP (=C)	Peru	South Am	n-rec	1973	Andigena	Renacimiento	MT708142
198	MT708143		I-2	S	DP (=D)	Peru	South Am	n-rec	1973	S. goniocalyx	Runtush	MT708143
199	MT752611	1	II-3	G	Apu008	Peru-Apu	South Am	n-rec	2018	Solanum ? chaucha	Putis	MT752611
200	MT752612	2	I-1	C	Apu010A	Peru-Apu	South Am	n-rec	2018	Solanum ? chaucha		MT752612
201	MT752613	3	II-3	G	Apu100A	Peru-Apu	South Am	n-rec	2018	Solanum ? chaucha		MT752613
202	MT752614	4	II-3	G	Cca004-1	Peru-Cca	South Am	n-rec	2016	(Monserrate x Atzimba) x a mixture of early clones	Amarilis	MT752614
203	MT752615	5	I-1		Cca004-2	Peru-Cca	South Am	rec	2016	mix		MT752615
204	MT752616	6	II-3	G	Cca005	Peru-Cca	South Am	n-rec	2016	tuberosum x andigena	Perricholi	MT752616
205	MT752617	7	I-1	C	Cca006	Peru-Cca	South Am	n-rec	2016	tuberosum x andigena		MT752617
206	MT752618	8	I-1	D	Cca008	Peru-Cca	South Am	n-rec	2016	(Monserrate x Atzimba) x a mixture of early clones	Amarilis	MT752618
207	MT752619	9	I-1	C	Cca009	Peru-Cca	South Am	n-rec	2016	mix		MT752619
208	MT752620	10	I-1	C	Cca010	Peru-Cca	South Am	n-rec	2016	tuberosum x andigena	Perricholi	
209	MT752621	11	I-1	D	Cca010B	Peru-Cca	South Am	n-rec	2016	(Monserrate x Atzimba) x a mixture of early clones	Amarilis	MT752621
210	MT752622	12	II-3	G	Cca012	Peru-Cca	South Am	n-rec	2016	S. phureja	Chaucha	MT752622
211	MT752623	13	II-3	H	Cca016	Peru-Cca	South Am	n-rec	2016	S. phureja		MT752623
212	MT752624	14	II-3	G	Cca018	Peru-Cca	South Am	n-rec	2016	S. phureja		MT752624
213	MT752625	15	II-3	I	Cca020	Peru-Cca	South Am	n-rec	2016	tuberosum x andigena		MT752625

214	MT752626	16	I-1	A	Cca020B	Peru-Cca	South Am	n-rec	2016	S. phureja		MT752626
215	MT752627	17	I-1	D	Cca022	Peru-Cca	South Am	n-rec	2016	(Monserrate x Atzimba) x a mixture of early clones	Amarilis	MT752627
216	MT752628	18	I-1	S	Cca035	Peru-Cca	South Am	n-rec	2016	Atzimba x A1(316) x (Phureja 1386 x Phureja 1339) x Kathadin	Molinera	MT752628
217	MT752629	19	I-1	S	Cca037	Peru-Cca	South Am	n-rec	2016	Atzimba x A1(316) x (Phureja 1386 x Phureja 1339) x Kathadin		
218	MT752630	20	II-3	G	Cca041	Peru-Cca	South Am	n-rec	2016	(Monserrate x Atzimba) x a mixture of early clones	Amarilis	MT752630
219	MT752631	21	I-1		Cca043	Peru-Cca	South Am	rec	2016	mix		MT752631
220	MT752632	22	I-1	A	Cca046B	Peru-Cca	South Am	n-rec	2016	mix		
221	MT752633	23	I-1	A	Cca047	Peru-Cca	South Am	n-rec	2016	mix		MT752633
222	MT752634	24	I-1	A	Cca048	Peru-Cca	South Am	n-rec	2016	mix		MT752634
223	MT752635	25	I-1	D	Cca049	Peru-Cca	South Am	n-rec	2016	mix		MT752635
224	MT752636	26	I-1	C	Cca051	Peru-Cca	South Am	n-rec	2016	tuberosum x andigena	Yungay	
225	MT752637	27	I-1	C	Cca052	Peru-Cca	South Am	n-rec	2016	tuberosum x andigena		
226	MT752638	28	I-1	D	Cca053	Peru-Cca	South Am	n-rec	2016	tuberosum x andigena		MT752638
227	MT752639	29	I-1	C	Cca055	Peru-Cca	South Am	n-rec	2016	tuberosum x andigena		MT752639
228	MT752640	30	I-1	C	Cca056	Peru-Cca	South Am	n-rec	2016	tuberosum x andigena		
229	MT752641	31	I-1	C	Cca058	Peru-Cca	South Am	n-rec	2016	tuberosum x andigena		

230	MT752642	32	I-1	C	Cca067	Peru-Cca	South Am	n-rec	2016	tuberosum x andigena	Mix (Canchan and Amarilis)	MT752642
231	MT752643	33	I-1	D	Cca068	Peru-Cca	South Am	n-rec	2016	tuberosum x andigena		MT752643
232	MT752644	34	I-1	A	Cca069	Peru-Cca	South Am	n-rec	2016	tuberosum x andigena		MT752644
233	MT752645	35	I-1	A	Cca070	Peru-Cca	South Am	n-rec	2016	tuberosum x andigena		MT752645
234	MT752646	36	I-1	A	Cca070B	Peru-Cca	South Am	n-rec	2016	tuberosum x andigena		MT752646
235	MT752647	37	II-3	G	Cca070C	Peru-Cca	South Am	n-rec	2016	tuberosum x andigena		MT752647
236	MT752648	38	I-1	A	Cca071-1	Peru-Cca	South Am	n-rec	2016	S. phureja	Chaucha	MT752648
237	MT752649	39	I-1	A	Cca071-2	Peru-Cca	South Am	n-rec	2016	S. phureja		
238	MT752650	40	I-1	A	Cca072	Peru-Cca	South Am	n-rec	2016	S. phureja		MT752650
239	MT752651	41	I-1	A	Cca075-1	Peru-Cca	South Am	n-rec	2016	S. phureja		MT752651
240	MT752652	42	I-1	A	Cca075-2	Peru-Cca	South Am	n-rec	2016	S. phureja		MT752652
241	MT752653	43	I-1	B	Cca078	Peru-Cca	South Am	n-rec	2016	S. phureja		MT752653
242	MT752654	44	I-1	B	Cca080E	Peru-Cca	South Am	n-rec	2016	S. phureja		MT752654
243	MT752655	45	I-1	A	Cca083	Peru-Cca	South Am	n-rec	2016	tuberosum x andigena	Yungay	MT752655
244	MT752656	46	I-1	C	Cca085	Peru-Cca	South Am	n-rec	2016	tuberosum x andigena		MT752656
245	MT752657	47	I-1	B	Cca094	Peru-Cca	South Am	n-rec	2016	tuberosum x andigena	Mix (Chaucha and Amarilis)	MT752657
246	MT752658	48	I-1	A	Cca096	Peru-Cca	South Am	n-rec	2016	tuberosum x andigena		MT752658
247	MT752659	49	II-3	G	Cca099	Peru-Cca	South Am	n-rec	2016	tuberosum x andigena		MT752659
248	MT752660	50	II-3	G	Cca100	Peru-Cca	South Am	n-rec	2016	tuberosum x andigena		MT752660
249	MT752661	51	II-3	H	Cca100B	Peru-Cca	South Am	n-rec	2016	tuberosum x andigena		MT752661
250	MT752662	52	I-1	A	Cca102	Peru-Cca	South Am	n-rec	2016	S. phureja	Chaucha	MT752662
251	MT752663	53	II-2	F	Cca104-1	Peru-Cca	South Am	n-rec	2016	S. phureja		MT752663
252	MT752664	54	II-3	G	Cca104-2	Peru-Cca	South Am	n-rec	2016	S. phureja		MT752664

253	MT752665	55	II-3	G	Cca107	Peru-Cca	South Am	n-rec	2016	S. phureja		MT752665
254	MT752666	56	II-3	H	Cca110C	Peru-Cca	South Am	n-rec	2016	S. phureja		MT752666
255	MT752667	57	II-3	H	Cca111	Peru-Cca	South Am	n-rec	2016	S. phureja		MT752667
256	MT752668	58	I-1	A	Cca118	Peru-Cca	South Am	n-rec	2016	S. phureja		MT752668
257	MT752669	59	I-1	A	Cca119-1	Peru-Cca	South Am	n-rec	2016	S. phureja		MT752669
258	MT752670	60	I-1	A	Cca119-2	Peru-Cca	South Am	n-rec	2016	S. phureja		
259	MT752671	61	I-1	A	Cca120	Peru-Cca	South Am	n-rec	2016	S. phureja		MT752671
260	MT752672	62	I-1	A	Cca120B	Peru-Cca	South Am	n-rec	2016	S. phureja		MT752672
261	MT752673	63	II-3	G	Cca122-1	Peru-Cca	South Am	n-rec	2016	tuberosum x andigena	Yungay	MT752673
262	MT752674	64	I-1	B	Cca122-2	Peru-Cca	South Am	n-rec	2016	tuberosum x andigena		MT752674
263	MT752675	65	II-2	F	Cca123	Peru-Cca	South Am	n-rec	2016	tuberosum x andigena		MT752675
264	MT752676	66	I-1	B	Cca125-1	Peru-Cca	South Am	n-rec	2016	tuberosum x andigena		MT752676
265	MT752677	67	I-1	B	Cca125-2	Peru-Cca	South Am	n-rec	2016	tuberosum x andigena		MT752677
266	MT752678	68	II-3	G	Cca126	Peru-Cca	South Am	n-rec	2016	tuberosum x andigena		MT752678
267	MT752679	69	II-3	G	Cca128	Peru-Cca	South Am	n-rec	2016	tuberosum x andigena		
268	MT752680	70	II-3	G	Cca130C	Peru-Cca	South Am	n-rec	2016	tuberosum x andigena		
269	MT752681	71	II-3	G	Cus009	Peru-Cus	South Am	n-rec	2016	tuberosum x andigena	Yungay	MT752681
270	MT752682	72	II-3	G	Cus010	Peru-Cus	South Am	n-rec	2016	tuberosum x andigena		MT752682
271	MT752683	73	II-3	G	Cus016-1	Peru-Cus	South Am	n-rec	2016	S. tuberosum ssp. tuberosum x	Cica	MT752683
272	MT752684	74	I-1	C	Cus016-2	Peru-Cus	South Am	n-rec	2016	Andigena		MT752684
273	MT752685	75	II-2	S	Cus075	Peru-Cus	South Am	n-rec	2016	Andigena	Ccompis	MT752685
274	MT752686	76	II-3	G	Cus086	Peru-Cus	South Am	n-rec	2016	Andigena		MT752686
275	MT752687	77	II-3	G	Cus088	Peru-Cus	South Am	n-rec	2016	Andigena		MT752687

276	MT752688	78	II-3	G	Cus089-1	Peru-Cus	South Am	n-rec	2016	Andigena		MT752688
277	MT752689	79	I-1		Cus089-2	Peru-Cus	South Am	rec	2016	Andigena		MT752689
278	MT752690	80	I-1	B	Cus089-3	Peru-Cus	South Am	n-rec	2016	Andigena		
279	MT752691	81	I-1	B	Cus122	Peru-Cus	South Am	n-rec	2016	tuberosum x andigena	Yungay	MT752691
280	MT752692	82	II-3	G	Cus127	Peru-Cus	South Am	n-rec	2016	tuberosum x andigena		MT752692
281	MT752693	83	II-3	I	Cus130B	Peru-Cus	South Am	n-rec	2016	tuberosum x andigena		MT752693
282	MT752694	84	II-3	G	Hua001	Peru-Hua	South Am	n-rec	2016	tuberosum x andigena	Andina	MT752694
283	MT752695	85	II-3	G	Hua002	Peru-Hua	South Am	n-rec	2016	tuberosum x andigena		MT752695
284	MT752696	86	II-3	G	Hua012	Peru-Hua	South Am	n-rec	2016	tuberosum x andigena	Yungay	MT752696
285	MT752697	87	I-1	A	Hua013	Peru-Hua	South Am	n-rec	2016	tuberosum x andigena		MT752697
286	MT752698	88	II-3	G	Hua016	Peru-Hua	South Am	n-rec	2016	tuberosum x andigena		MT752698
287	MT752699	89	II-3	G	Hua017	Peru-Hua	South Am	n-rec	2016	tuberosum x andigena		MT752699
288	MT752700	90	II-3	G	Hua020	Peru-Hua	South Am	n-rec	2016	tuberosum x andigena		MT752700
289	MT752701	91	I-1	A	Hua021	Peru-Hua	South Am	n-rec	2018	tuberosum x andigena	Mix (Ccompis and Huayro)	MT752701
290	MT752702	92	II-3	G	Hua026	Peru-Hua	South Am	n-rec	2018	tuberosum x andigena		MT752702
291	MT752703	93	I-1	A	Hua051	Peru-Hua	South Am	n-rec	2018	tuberosum x andigena	unknown	
292	MT752704	94	II-3	G	Hua055-1	Peru-Hua	South Am	n-rec	2018	tuberosum x andigena		MT752704
293	MT752705	95	I-1	S	Hua055-2	Peru-Hua	South Am	n-rec	2018	tuberosum x andigena		MT752705
294	MT752706	96	II-3	G	Hua057-1	Peru-Hua	South Am	n-rec	2018	tuberosum x andigena		MT752706
295	MT752707	97	I-1	S	Hua057-2	Peru-Hua	South Am	n-rec	2018	tuberosum x andigena		
296	MT752708	98	II-3	G	Hua059	Peru-Hua	South Am	n-rec	2018	tuberosum x andigena		MT752708

297	MT752709	99	II-3	G	Hua060	Peru-Hua	South Am	n-rec	2018	tuberosum x andigena		MT752709
298	MT752710	100	II-2	F	Hua060A	Peru-Hua	South Am	n-rec	2018	tuberosum x andigena		MT752710
299	MT752711	101	I-1	A	Hco001	Peru-Hco	South Am	n-rec	2016	tuberosum x andigena	Mix (Yungay and Amarilis)	MT752711
300	MT752712	102	II-2	F	Hco002-1	Peru-Hco	South Am	n-rec	2016	tuberosum x andigena		MT752712
301	MT752713	103	I-1	C	Hco002-2	Peru-Hco	South Am	n-rec	2016	tuberosum x andigena		MT752713
302	MT752714	104	I-1	D	Hco004	Peru-Hco	South Am	n-rec	2016	tuberosum x andigena		MT752714
303	MT752715	105	II-3	S	Hco005B	Peru-Hco	South Am	n-rec	2016	tuberosum x andigena		MT752715
304	MT752716	106	I-1	C	Hco007	Peru-Hco	South Am	n-rec	2016	tuberosum x andigena		MT752716
305	MT752717	107	I-1	A	Hco012	Peru-Hco	South Am	n-rec	2016	tuberosum x andigena	Yungay	
306	MT752718	108	II-3	S	Hco013	Peru-Hco	South Am	n-rec	2016	tuberosum x andigena		MT752718
307	MT752719	109	I-1	A	Hco015	Peru-Hco	South Am	n-rec	2016	tuberosum x andigena		
308	MT752720	110	I-1	A	Hco016	Peru-Hco	South Am	n-rec	2016	tuberosum x andigena		MT752720
309	MT752721	111	I-1	A	Hco019	Peru-Hco	South Am	n-rec	2016	tuberosum x andigena		MT752721
310	MT752722	112	I-1	B	Hco021-1	Peru-Hco	South Am	n-rec	2016	tuberosum x andigena	Mix (Canchan and Blanca)	MT752722
311	MT752723	113	I-1	B	Hco021-2	Peru-Hco	South Am	n-rec	2016	tuberosum x andigena		MT752723
312	MT752724	114	I-1	A	Hco022	Peru-Hco	South Am	n-rec	2016	tuberosum x andigena		MT752724
313	MT752725	115	I-1	C	Hco023	Peru-Hco	South Am	n-rec	2016	tuberosum x andigena		MT752725
314	MT752726	116	II-3	G	Hco025	Peru-Hco	South Am	n-rec	2016	tuberosum x andigena		
315	MT752727	117	II-2	F	Hco026-1	Peru-Hco	South Am	n-rec	2016	tuberosum x andigena		MT752727
316	MT752728	118	I-1	C	Hco026-2	Peru-Hco	South Am	n-rec	2016	tuberosum x andigena		MT752728
317	MT752729	119	II-2		Hco027-1	Peru-Hco	South Am	rec	2016	tuberosum x andigena		
318	MT752730	120	II-2		Hco027-2	Peru-Hco	South Am	rec	2016	tuberosum x andigena		MT752730
319	MT752731	121	I-1	A	Hco028	Peru-Hco	South Am	n-rec	2016	tuberosum x andigena		MT752731

320	MT752732	122	I-1	B	Hco029	Peru-Hco	South Am	n-rec	2016	tuberosum x andigena		MT752732
321	MT752733	123	I-1	B	Hco030B	Peru-Hco	South Am	n-rec	2016	tuberosum x andigena		MT752733
322	MT752734	124	I-1	C	Hco031	Peru-Hco	South Am	n-rec	2016	tuberosum x andigena	Yungay	MT752734
323	MT752735	125	I-1	A	Hco032	Peru-Hco	South Am	n-rec	2016	tuberosum x andigena		MT752735
324	MT752736	126	I-1	A	Hco033	Peru-Hco	South Am	n-rec	2016	tuberosum x andigena		MT752736
325	MT752737	127	I-1	A	Hco034	Peru-Hco	South Am	n-rec	2016	tuberosum x andigena		MT752737
326	MT752738	128	II-3	G	Hco035	Peru-Hco	South Am	n-rec	2016	tuberosum x andigena		MT752738
327	MT752739	129	I-1	A	Hco036	Peru-Hco	South Am	n-rec	2016	tuberosum x andigena		MT752739
328	MT752740	130	II-3	G	Hco038-1	Peru-Hco	South Am	n-rec	2016	tuberosum x andigena		MT752740
329	MT752741	131	I-1	C	Hco038-2	Peru-Hco	South Am	n-rec	2016	tuberosum x andigena		MT752741
330	MT752742	132	II-3	G	Hco039-1	Peru-Hco	South Am	n-rec	2016	tuberosum x andigena		MT752742
331	MT752743	133	I-1	S	Hco039-2	Peru-Hco	South Am	n-rec	2016	tuberosum x andigena		MT752743
332	MT752744	134	II-3	G	Hco054	Peru-Hco	South Am	n-rec	2016	tuberosum x andigena		MT752744
333	MT752745	135	II-3	K	Hco056	Peru-Hco	South Am	n-rec	2016	tuberosum x andigena		MT752745
334	MT752746	136	II-2	F	Hco057	Peru-Hco	South Am	n-rec	2016	tuberosum x andigena		MT752746
335	MT752747	137	II-3	G	Hco058	Peru-Hco	South Am	n-rec	2016	tuberosum x andigena		MT752747
336	MT752748	138	II-3	G	Hco059-1	Peru-Hco	South Am	n-rec	2016	tuberosum x andigena		MT752748
337	MT752749	139	I-1	A	Hco059-2	Peru-Hco	South Am	n-rec	2016	tuberosum x andigena		MT752749
338	MT752750	140	I-1	A	Hco062	Peru-Hco	South Am	n-rec	2016	tuberosum x andigena	Yungay	MT752750
339	MT752751	141	I-1	D	Hco066	Peru-Hco	South Am	n-rec	2016	tuberosum x andigena		MT752751
340	MT752752	142	I-1	A	Hco067	Peru-Hco	South Am	n-rec	2016	tuberosum x andigena		MT752752
341	MT752753	143	I-1	A	Hco068	Peru-Hco	South Am	n-rec	2016	tuberosum x andigena		MT752753
342	MT752754	144	II-3	L	Hco070	Peru-Hco	South Am	n-rec	2016	tuberosum x andigena		MT752754

343	MT752755	145	I-1	A	Ica011	Peru-Ica	South Am	n-rec	2017	[{(tbr x S. raphanifolium) x tbr} x adg] x tbr	Unica	
344	MT752756	146	II-3	G	Ica012	Peru-Ica	South Am	n-rec	2017	[{(tbr x S. raphanifolium) x tbr} x adg] x tbr		MT752756
345	MT752757	147	II-3	K	Ica016	Peru-Ica	South Am	n-rec	2017	[{(tbr x S. raphanifolium) x tbr} x adg] x tbr		MT752757
346	MT752758	148	II-3		Ica017-1	Peru-Ica	South Am	rec	2017	[{(tbr x S. raphanifolium) x tbr} x adg] x tbr		MT752758
347	MT752759	149	I-1	A	Ica017-2	Peru-Ica	South Am	n-rec	2017	[{(tbr x S. raphanifolium) x tbr} x adg] x tbr		MT752759
348	MT752760	150	II-3	G	Ica024	Peru-Ica	South Am	n-rec	2017	tuberosum	Canchan	MT752760
349	MT752761	151	II-3		Ica027-1	Peru-Ica	South Am	rec	2017	Andigena		MT752761
350	MT752762	152	I-1		Ica027-2	Peru-Ica	South Am	rec	2017	Andigena		MT752762
351	MT752763	153	I-1		Ica027-3	Peru-Ica	South Am	rec	2017	Andigena		
352	MT752764	154	II-3	G	Ica028	Peru-Ica	South Am	n-rec	2017	Andigena		MT752764
353	MT752765	155	II-3	G	Ica029	Peru-Ica	South Am	n-rec	2017	Andigena		MT752765
354	MT752766	156	II-3	J	Ica030A	Peru-Ica	South Am	n-rec	2017	Andigena		MT752766
355	MT752767	157	II-3	K	Ica031-1	Peru-Ica	South Am	n-rec	2017	Andigena		
356	MT752768	158	II-3	K	Ica031-2	Peru-Ica	South Am	n-rec	2017	Andigena		MT752768
357	MT752769	159	II-2	F	Ica035	Peru-Ica	South Am	n-rec	2017	Andigena		MT752769
358	MT752770	160	II-2	F	Ica036	Peru-Ica	South Am	n-rec	2017	Andigena		MT752770
359	MT752771	161	I-1	A	Ica037	Peru-Ica	South Am	n-rec	2017	Andigena		MT752771
360	MT752772	162	II-2	F	Ica039	Peru-Ica	South Am	n-rec	2017	Andigena		MT752772
361	MT752773	163	II-3	G	Ica040	Peru-Ica	South Am	n-rec	2017	Andigena		MT752773
362	MT752774	164	II-3	G	Ica040A	Peru-Ica	South Am	n-rec	2017	Andigena		MT752774
363	MT752775	165	II-3	G	Ica045	Peru-Ica	South Am	n-rec	2017	Andigena		MT752775
364	MT752776	166	II-3	G	Ica049	Peru-Ica	South Am	n-rec	2017	Andigena		MT752776

365	MT752777	167	I-1	D	Ica052	Peru-Ica	South Am	n-rec	2017	[{(tbr x S. raphanifolium) x tbr} x adg] x tbr	Unica	MT752777
366	MT752778	168	II-3	G	Ica055	Peru-Ica	South Am	n-rec	2017	[{(tbr x S. raphanifolium) x tbr} x adg] x tbr		MT752778
367	MT752779	169	I-1	C	Ica091	Peru-Ica	South Am	n-rec	2017	tuberosum x andigena	Yungay	MT752779
368	MT752780	170	II-3	G	Ica095	Peru-Ica	South Am	n-rec	2017	tuberosum x andigena		MT752780
369	MT752781	171	II-3	G	Ica096-1	Peru-Ica	South Am	n-rec	2017	tuberosum x andigena		
370	MT752782	172	I-1	C	Ica096-2	Peru-Ica	South Am	n-rec	2017	tuberosum x andigena		MT752782
371	MT752783	173	II-3		Ica098-1	Peru-Ica	South Am	rec	2017	tuberosum x andigena		MT752783
372	MT752784	174	I-1	B	Ica098-2	Peru-Ica	South Am	n-rec	2017	tuberosum x andigena		MT752784
373	MT752785	175	II-2	F	Ica099-1	Peru-Ica	South Am	n-rec	2017	tuberosum x andigena		MT752785
374	MT752786	176	I-1	A	Ica099-2	Peru-Ica	South Am	n-rec	2017	tuberosum x andigena		MT752786
375	MT752787	177	I-1	A	Ica100	Peru-Ica	South Am	n-rec	2017	tuberosum x andigena		MT752787
376	MT752788	178	II-2	F	Jin014	Peru-Jin	South Am	n-rec	2016	tuberosum x andigena	Yungay	MT752788
377	MT752789	179	II-3	G	Jin017	Peru-Jin	South Am	n-rec	2016	tuberosum x andigena		MT752789
378	MT752790	180	II-3	L	Jin032	Peru-Jin	South Am	n-rec	2016	tuberosum x andigena		MT752790
379	MT752791	181	I-1	A	Jin035	Peru-Jin	South Am	n-rec	2016	tuberosum x andigena		MT752791
380	MT752792	182	II-3	G	Jin041	Peru-Jin	South Am	n-rec	2016	[{(tbr x S. raphanifolium) x tbr} x adg] x tbr	Unica	MT752792
381	MT752793	183	II-3	L	Jin042-1	Peru-Jin	South Am	n-rec	2016	[{(tbr x S. raphanifolium) x tbr} x adg] x tbr		MT752793
382	MT752794	184	II-3	L	Jin042-2	Peru-Jin	South Am	n-rec	2016	[{(tbr x S. raphanifolium) x tbr} x adg] x tbr		
383	MT752795	185	II-2	F	Jin043	Peru-Jin	South Am	n-rec	2016	[{(tbr x S. raphanifolium) x tbr} x adg] x tbr		MT752795
384	MT752796	186	II-3	G	Jin045	Peru-Jin	South Am	n-rec	2016	[{(tbr x S. raphanifolium) x tbr} x adg] x tbr		

385	MT752797	187	I-1	A	Jin046	Peru-Jin	South Am	n-rec	2016	[{(tbr x S. raphanifolium) x tbr} x adg] x tbr		MT752797
386	MT752798	188	II-3	G	Jin048	Peru-Jin	South Am	n-rec	2016	[{(tbr x S. raphanifolium) x tbr} x adg] x tbr		MT752798
387	MT752799	189	II-3	K	Jin051	Peru-Jin	South Am	n-rec	2016	[{(tbr x S. raphanifolium) x tbr} x adg] x tbr		MT752799
388	MT752800	190	II-3	G	Jin054	Peru-Jin	South Am	n-rec	2016	[{(tbr x S. raphanifolium) x tbr} x adg] x tbr		MT752800
389	MT752801	191	II-2	F	Jin056	Peru-Jin	South Am	n-rec	2016	[{(tbr x S. raphanifolium) x tbr} x adg] x tbr		MT752801
390	MT752802	192	II-2	F	Jin057-1	Peru-Jin	South Am	n-rec	2016	[{(tbr x S. raphanifolium) x tbr} x adg] x tbr		MT752802
391	MT752803	193	II-3	G	Jin057-2	Peru-Jin	South Am	n-rec	2016	[{(tbr x S. raphanifolium) x tbr} x adg] x tbr		MT752803
392	MT752804	194	II-2	F	Jin059	Peru-Jin	South Am	n-rec	2016	[{(tbr x S. raphanifolium) x tbr} x adg] x tbr		
393	MT752805	195	II-3	G	Jin061	Peru-Jin	South Am	n-rec	2016	tuberosum x andigena	Yungay	MT752805
394	MT752806	196	II-3	G	Jin075	Peru-Jin	South Am	n-rec	2016	[{(tbr x S. raphanifolium) x tbr} x adg] x tbr	Unica	MT752806
395	MT752807	197	II-3	G	Jin089	Peru-Jin	South Am	n-rec	2016	tuberosum x andigena	Perricholi	
396	MT752808	198	I-1	A	Jin090	Peru-Jin	South Am	n-rec	2016	tuberosum x andigena		MT752808
397	MT752809	199	II-3	J	Jin091	Peru-Jin	South Am	n-rec	2016	tuberosum x andigena	Andina	MT752809
398	MT752810	200	II-3	G	Jin095	Peru-Jin	South Am	n-rec	2016	tuberosum x andigena		MT752810
399	MT752811	201	II-3	J	Jin099	Peru-Jin	South Am	n-rec	2016	tuberosum x andigena		MT752811
400	MT752812	202	II-3	G	Jin101-1	Peru-Jin	South Am	n-rec	2016	tuberosum x andigena	Yungay	MT752812
401	MT752813	203	I-1	B	Jin101-2	Peru-Jin	South Am	n-rec	2016	tuberosum x andigena		MT752813
402	MT752814	204	I-1	C	Jin102	Peru-Jin	South Am	n-rec	2016	tuberosum x andigena		MT752814
403	MT752815	205	I-1	C	Jin103	Peru-Jin	South Am	n-rec	2016	tuberosum x andigena		MT752815
404	MT752816	206	I-1	B	Jin104	Peru-Jin	South Am	n-rec	2016	tuberosum x andigena		MT752816

405	MT752817	207	II-3	I	Jin105-1	Peru-Jin	South Am	n-rec	2016	tuberosum x andigena		MT752817
406	MT752818	208	I-1	B	Jin105-2	Peru-Jin	South Am	n-rec	2016	tuberosum x andigena		
407	MT752819	209	II-3	H	Jin106-1	Peru-Jin	South Am	n-rec	2016	tuberosum x andigena		MT752819
408	MT752820	210	I-1	B	Jin106-2	Peru-Jin	South Am	n-rec	2016	tuberosum x andigena		MT752820
409	MT752821	211	I-1	S	Jin107	Peru-Jin	South Am	n-rec	2016	tuberosum x andigena		MT752821
410	MT752822	212	I-1	C	Jin108	Peru-Jin	South Am	n-rec	2016	tuberosum x andigena		MT752822
411	MT752823	213	II-3	G	Jin109-1	Peru-Jin	South Am	n-rec	2016	tuberosum x andigena		MT752823
412	MT752824	214	I-1	C	Jin109-2	Peru-Jin	South Am	n-rec	2016	tuberosum x andigena		MT752824
413	MT752825	215	II-3	I	Jin110	Peru-Jin	South Am	n-rec	2016	tuberosum x andigena		MT752825
414	MT752826	216	I-1	S	Jin110B	Peru-Jin	South Am	n-rec	2016	tuberosum x andigena		MT752826
415	MT752827	217	I-1	A	Jin111	Peru-Jin	South Am	n-rec	2016	tuberosum x andigena		MT752827
416	MT752828	218	I-1	B	Jin112	Peru-Jin	South Am	n-rec	2016	tuberosum x andigena		MT752828
417	MT752829	219	II-3	I	Jin113	Peru-Jin	South Am	n-rec	2016	tuberosum x andigena		MT752829
418	MT752830	220	I-1	B	Jin114	Peru-Jin	South Am	n-rec	2016	tuberosum x andigena		MT752830
419	MT752831	221	I-1	A	Jin116	Peru-Jin	South Am	n-rec	2016	tuberosum x andigena		MT752831
420	MT752832	222	II-3	G	Jin118-1	Peru-Jin	South Am	n-rec	2016	tuberosum x andigena		MT752832
421	MT752833	223	I-1	A	Jin118-2	Peru-Jin	South Am	n-rec	2016	tuberosum x andigena		MT752833
422	MT752834	224	I-1	B	Jin119	Peru-Jin	South Am	n-rec	2016	tuberosum x andigena		MT752834
423	MT752835	225	I-1	B	Jin120	Peru-Jin	South Am	n-rec	2016	tuberosum x andigena		MT752835
424	MT752836	226	I-1	A	Jin120B	Peru-Jin	South Am	n-rec	2016	tuberosum x andigena		MT752836
425	MT752837	227	I-1	A	Jin122	Peru-Jin	South Am	n-rec	2016	tuberosum x andigena	Perricholi	MT752837
426	MT752838	228	II-3	J	Jin124	Peru-Jin	South Am	n-rec	2016	andigena		MT752838
427	MT752839	229	I-1	B	Jin125	Peru-Jin	South Am	n-rec	2016	andigena		MT752839
428	MT752840	230	I-1	B	Jin126	Peru-Jin	South Am	n-rec	2016	tuberosum x andigena	Canchan	MT752840

429	MT752841	231	I-1	A	Jin128	Peru-Jin	South Am	n-rec	2016	Andigena		MT752841
430	MT752842	232	II-3	G	Jin129	Peru-Jin	South Am	n-rec	2016	Andigena		MT752842
431	MT752843	233	I-1	A	Jin130	Peru-Jin	South Am	n-rec	2016	Andigena		MT752843
432	MT752844	234	I-1	A	Jin130B	Peru-Jin	South Am	n-rec	2016	Andigena		
433	MT752845	235	I-1	A	Jin131	Peru-Jin	South Am	n-rec	2016	Andigena	Mix (Yungan and Canchan)	MT752845
434	MT752846	236	I-1	C	Jin132	Peru-Jin	South Am	n-rec	2016	Andigena		MT752846
435	MT752847	237	I-1	A	Jin133	Peru-Jin	South Am	n-rec	2016	Andigena		MT752847
436	MT752848	238	II-3	H	Jin134-1	Peru-Jin	South Am	n-rec	2016	Andigena		MT752848
437	MT752849	239	I-1	A	Jin134-2	Peru-Jin	South Am	n-rec	2016	Andigena		MT752849
438	MT752850	240	I-1	A	Jin137	Peru-Jin	South Am	n-rec	2016	Andigena		MT752850
439	MT752851	241	I-1	A	Jin139-1	Peru-Jin	South Am	n-rec	2016	Andigena		MT752851
440	MT752852	242	I-1	A	Jin139-2	Peru-Jin	South Am	n-rec	2016	Andigena		
441	MT752853	243	I-1	A	Jin140	Peru-Jin	South Am	n-rec	2016	Andigena		MT752853
442	MT752854	244	II-3	H	Jin161-1	Peru-Jin	South Am	n-rec	2016	tuberosum x andigena	Yungay	MT752854
443	MT752855	245	I-1	C	Jin161-2	Peru-Jin	South Am	n-rec	2016	tuberosum x andigena		MT752855
444	MT752856	246	I-1	A	Jin162	Peru-Jin	South Am	n-rec	2016	tuberosum x andigena		MT752856
445	MT752857	247	I-1		Jin163	Peru-Jin	South Am	rec	2016	tuberosum x andigena		MT752857
446	MT752858	248	II-2	F	Jin164	Peru-Jin	South Am	n-rec	2016	tuberosum x andigena		MT752858
447	MT752859	249	II-3	G	Jin166	Peru-Jin	South Am	n-rec	2016	tuberosum x andigena		MT752859
448	MT752860	250	II-3	H	Jin168	Peru-Jin	South Am	n-rec	2016	tuberosum x andigena		MT752860
449	MT752861	251	II-3	G	Jin169	Peru-Jin	South Am	n-rec	2016	tuberosum x andigena		MT752861
450	MT752862	252	II-3	G	Jin170-1	Peru-Jin	South Am	n-rec	2016	tuberosum x andigena		MT752862
451	MT752863	253	I-1	A	Jin170-2	Peru-Jin	South Am	n-rec	2016	tuberosum x andigena		MT752863
452	MT752864	254	I-1	A	Jin170B	Peru-Jin	South Am	n-rec	2016	tuberosum x andigena		MT752864

453	MT752865	255	II-3	G	Jin171-1	Peru-Jin	South Am	n-rec	2016	tuberosum x andigena	Yungay	MT752865
454	MT752866	256	I-1	B	Jin171-2	Peru-Jin	South Am	n-rec	2016	tuberosum x andigena		MT752866
455	MT752867	257	II-3	G	Jin172-1	Peru-Jin	South Am	n-rec	2016	tuberosum x andigena		MT752867
456	MT752868	258	I-1	B	Jin172-2	Peru-Jin	South Am	n-rec	2016	tuberosum x andigena		MT752868
457	MT752869	259	I-1		Jin173	Peru-Jin	South Am	rec	2016	tuberosum x andigena		MT752869
458	MT752870	260	II-2	F	Jin174-1	Peru-Jin	South Am	n-rec	2016	tuberosum x andigena		MT752870
459	MT752871	261	II-3	G	Jin174-2	Peru-Jin	South Am	n-rec	2016	tuberosum x andigena		MT752871
460	MT752872	262	I-1	B	Jin174-3	Peru-Jin	South Am	n-rec	2016	tuberosum x andigena		MT752872
461	MT752873	263	I-1	B	Jin175	Peru-Jin	South Am	n-rec	2016	tuberosum x andigena		MT752873
462	MT752874	264	II-3	G	Jin176	Peru-Jin	South Am	n-rec	2016	tuberosum x andigena		MT752874
463	MT752875	265	II-3	G	Jin177-1	Peru-Jin	South Am	n-rec	2016	tuberosum x andigena		MT752875
464	MT752876	266	I-1	C	Jin177-2	Peru-Jin	South Am	n-rec	2016	tuberosum x andigena		MT752876
465	MT752877	267	I-1		Jin178	Peru-Jin	South Am	rec	2016	tuberosum x andigena		MT752877
466	MT752878	268	I-1	A	Jin179	Peru-Jin	South Am	n-rec	2016	tuberosum x andigena		MT752878
467	MT752879	269	I-1	A	Jin180	Peru-Jin	South Am	n-rec	2016	tuberosum x andigena		MT752879
468	MT752880	270	I-1	A	Jin180B	Peru-Jin	South Am	n-rec	2016	tuberosum x andigena		
469	MT752881	271	II-3	G	Lim001	Peru-Lim	South Am	n-rec	2017	[{(tbr x S. raphanifolium) x tbr} x adg] x tbr	Unica	MT752881
470	MT752882	272	II-3	G	Lim002	Peru-Lim	South Am	n-rec	2017	[{(tbr x S. raphanifolium) x tbr} x adg] x tbr		MT752882
471	MT752883	273	II-2	F	Lim003	Peru-Lim	South Am	n-rec	2017	[{(tbr x S. raphanifolium) x tbr} x adg] x tbr		MT752883
472	MT752884	274	II-2	F	Lim027	Peru-Lim	South Am	n-rec	2017	[{(tbr x S. raphanifolium) x tbr} x adg] x tbr		MT752884
473	MT752885	275	I-1	D	Lim033	Peru-Lim	South Am	n-rec	2017	tuberosum x andigena	Canchan	MT752885

474	MT752886	276	I-1	B	Lim041	Peru-Lim	South Am	n-rec	2017	[{(tbr x S. raphanifolium) x tbr} x adg] x tbr	Unica	MT752886
475	MT752887	277	II-3	G	Lim042	Peru-Lim	South Am	n-rec	2017	[{(tbr x S. raphanifolium) x tbr} x adg] x tbr		MT752887
476	MT752888	278	I-1	B	Lim043	Peru-Lim	South Am	n-rec	2017	[{(tbr x S. raphanifolium) x tbr} x adg] x tbr		MT752888
477	MT752889	279	I-1	B	Lim045	Peru-Lim	South Am	n-rec	2017	[{(tbr x S. raphanifolium) x tbr} x adg] x tbr		MT752889
478	MT752890	280	II-3	G	Lim046	Peru-Lim	South Am	n-rec	2017	[{(tbr x S. raphanifolium) x tbr} x adg] x tbr		MT752890
479	MT752891	281	II-3	G	Lim048	Peru-Lim	South Am	n-rec	2017	[{(tbr x S. raphanifolium) x tbr} x adg] x tbr		
480	MT752892	282	II-2	F	Lim050-1	Peru-Lim	South Am	n-rec	2017	[{(tbr x S. raphanifolium) x tbr} x adg] x tbr		MT752892
481	MT752893	283	I-1	A	Lim050-2	Peru-Lim	South Am	n-rec	2017	[{(tbr x S. raphanifolium) x tbr} x adg] x tbr		MT752893
482	MT752894	284	II-3	G	Lim050A	Peru-Lim	South Am	n-rec	2017	[{(tbr x S. raphanifolium) x tbr} x adg] x tbr		MT752894
483	MT752895	285	II-3	G	Lim071	Peru-Lim	South Am	n-rec	2017	[{(tbr x S. raphanifolium) x tbr} x adg] x tbr	Unica	MT752895
484	MT752896	286	II-2		Lim084	Peru-Lim	South Am	rec	2017	[{(tbr x S. raphanifolium) x tbr} x adg] x tbr		MT752896
485	MT752897	287	II-3	G	Lim086	Peru-Lim	South Am	n-rec	2017	[{(tbr x S. raphanifolium) x tbr} x adg] x tbr		MT752897
486	MT752898	288	I-1	B	Lim088	Peru-Lim	South Am	n-rec	2017	[{(tbr x S. raphanifolium) x tbr} x adg] x tbr		MT752898
487	MT752899	289	I-1	B	Lim089	Peru-Lim	South Am	n-rec	2017	[{(tbr x S. raphanifolium) x tbr} x adg] x tbr		
488	MT752900	290	II-2	F	Lim090	Peru-Lim	South Am	n-rec	2017	[{(tbr x S. raphanifolium) x tbr} x adg] x tbr		MT752900
489	MT752901	291	II-3	G	Lim091-1	Peru-Lim	South Am	n-rec	2017	[{(tbr x S. raphanifolium) x tbr} x adg] x tbr	Unica	MT752901
490	MT752902	292	I-1	B	Lim091-2	Peru-Lim	South Am	n-rec	2017	[{(tbr x S. raphanifolium) x tbr} x adg] x tbr		MT752902
491	MT752903	293	I-1	A	Lim092	Peru-Lim	South Am	n-rec	2017	[{(tbr x S. raphanifolium) x tbr} x adg] x tbr		MT752903
492	MT752904	294	II-2	F	Lim093	Peru-Lim	South Am	n-rec	2017	[{(tbr x S. raphanifolium) x tbr} x adg] x tbr		MT752904
493	MT752905	295	I-1	B	Lim094	Peru-Lim	South Am	n-rec	2017	[{(tbr x S. raphanifolium) x tbr} x adg] x tbr		MT752905

494	MT752906	296	II-3	G	Lim095	Peru-Lim	South Am	n-rec	2017	[{(tbr x S. raphanifolium) x tbr} x adg] x tbr		MT752906
495	MT752907	297	II-2	F	Lim096-1	Peru-Lim	South Am	n-rec	2017	[{(tbr x S. raphanifolium) x tbr} x adg] x tbr		MT752907
496	MT752908	298	II-3	G	Lim096-2	Peru-Lim	South Am	n-rec	2017	[{(tbr x S. raphanifolium) x tbr} x adg] x tbr		MT752908
497	MT752909	299	I-1	A	Lim096-3	Peru-Lim	South Am	n-rec	2017	[{(tbr x S. raphanifolium) x tbr} x adg] x tbr		MT752909
498	MT752910	300	II-2	F	Lim097-1	Peru-Lim	South Am	n-rec	2017	[{(tbr x S. raphanifolium) x tbr} x adg] x tbr		MT752910
499	MT752911	301	II-3	S	Lim097-2	Peru-Lim	South Am	n-rec	2017	[{(tbr x S. raphanifolium) x tbr} x adg] x tbr		MT752911
500	MT752912	302	I-1	B	Lim097-3	Peru-Lim	South Am	n-rec	2017	[{(tbr x S. raphanifolium) x tbr} x adg] x tbr		MT752912
501	MT752913	303	I-1	B	Lim098	Peru-Lim	South Am	n-rec	2017	[{(tbr x S. raphanifolium) x tbr} x adg] x tbr		MT752913
502	MT752914	304	II-3	G	Lim099-1	Peru-Lim	South Am	n-rec	2017	[{(tbr x S. raphanifolium) x tbr} x adg] x tbr		MT752914
503	MT752915	305	I-1	A	Lim099-2	Peru-Lim	South Am	n-rec	2017	[{(tbr x S. raphanifolium) x tbr} x adg] x tbr		MT752915
504	MT752916	306	II-3	G	Lim100-1	Peru-Lim	South Am	n-rec	2017	[{(tbr x S. raphanifolium) x tbr} x adg] x tbr		MT752916
505	MT752917	307	I-1	A	Lim100-2	Peru-Lim	South Am	n-rec	2017	[{(tbr x S. raphanifolium) x tbr} x adg] x tbr		MT752917
506	MT752918	308	II-2	F	Pun001-1	Peru-Pun	South Am	n-rec	2018	Andigena	Huaycha	MT752918
507	MT752919	309	I-1		Pun001-2	Peru-Pun	South Am	rec	2018	Andigena		MT752919
508	MT752920	310	I-1	C	Pun002-1	Peru-Pun	South Am	n-rec	2018	Andigena		MT752920
509	MT752921	311	I-1	C	Pun002-2	Peru-Pun	South Am	n-rec	2018	Andigena		
510	MT752922	312	I-1	C	Pun003-1	Peru-Pun	South Am	n-rec	2018	Andigena		MT752922
511	MT752923	313	I-1	C	Pun003-2	Peru-Pun	South Am	n-rec	2018	Andigena		MT752923
512	MT752924	314	I-1	A	Pun004	Peru-Pun	South Am	n-rec	2018	Andigena		MT752924
513	MT752925	315	I-1	A	Pun015	Peru-Pun	South Am	n-rec	2018	Andigena	Ccompis	MT752925
514	MT752926	316	II-3	G	Pun019	Peru-Pun	South Am	n-rec	2018	Andigena		MT752926
515	MT752927	317	I-1	S	Pun031	Peru-Pun	South Am	n-rec	2018	Andigena	Improved variety	MT752927
516	MT752928	318	II-3	G	Pun032-1	Peru-Pun	South Am	n-rec	2018	Andigena		MT752928
517	MT752929	319	I-1	C	Pun032-2	Peru-Pun	South Am	n-rec	2018	Andigena		MT752929
518	MT752930	320	II-2	F	Pun033-1	Peru-Pun	South Am	n-rec	2018	Andigena		MT752930
519	MT752931	321	II-3	G	Pun033-2	Peru-Pun	South Am	n-rec	2018	Andigena		MT752931

520	MT752932	322	II-3	G	Pun035-1	Peru-Pun	South Am	n-rec	2018	Andigena		MT752932
521	MT752933	323	I-1		Pun035-2	Peru-Pun	South Am	rec	2018	Andigena		MT752933
522	MT752934	324	I-1	S	Pun035-3	Peru-Pun	South Am	n-rec	2018	Andigena		MT752934
523	MT752935	325	I-1	A	Pun036	Peru-Pun	South Am	n-rec	2018	Andigena		MT752935
524	MT752936	326	II-2	F	Pun040	Peru-Pun	South Am	n-rec	2018	Andigena		
525	NC_011620		I-1	B	X3	NethUSA	Eurasia	n-rec	?1988	N.tab		NC_011620
526			I-1			China	Asia					U19790
527	X55802		II-2	F	CP	Argentina	Eurasia	n-rec	?1990	S.t.t.		
528			I-1			China	Asia					X65015
529	X72214		II-2	S	HB	Netherlands	Eurasia	n-rec	?2004	S.t.t.		X72214
530			II-1			UK	Eurasia					X88782
531	Z23256		II-2	S	HB	UK	Eurasia	n-rec	?1993	S.t.t.		
532			I-1			Estonia	Eurasia					Z29333
533			I-1			Estonia	Eurasia					Z29335
534			I-1			Argentina	South Am					Z34261

File S2. The Accession Codes of the potato virus X isolates in the different clusters shown in Fig. 2.

Cluster A (75)

MT752626, MT752632, MT752633, MT752634, MT752644, MT752645, MT752646, MT752648, MT752649, MT752650, MT752651, MT752652, MT752655, MT752658, MT752662, MT752668, MT752669, MT752670, MT752671, MT752672, MT752697, MT752701, MT752703, MT752711, MT752717, MT752719, MT752720, MT752721, MT752724, MT752731, MT752735, MT752736, MT752737, MT752739, MT752749, MT752750, MT752752, MT752753, MT752755, MT752759, MT752771, MT752786, MT752787, MT752791, MT752797, MT752808, MT752827, MT752831, MT752833, MT752836, MT752837, MT752841, MT752843, MT752844, MT752845, MT752847, MT752849, MT752850, MT752851, MT752852, MT752853, MT752856, MT752863, MT752864, MT752878, MT752879, MT752880, MT752893, MT752903, MT752909, MT752915, MT752917, MT752924, MT752925, MT752935;

Cluster B (76)
AB056718, AB056719, AB195999, AB196000, AB196001, AF111193, AF272736, AF373782, D00344, EF423572, EU021215, EU571480, FJ461343, HQ450388, JF430080, KF575174, KF575175, KM659859, KR605396, KR605397, KU586452, KU586453, M38480, M72416, M95516, MF405302, MF682526, MF682527, MF682528, MH069212, MH282866, MK558273, MN125073, MT264741, MT374179, MT520804, MT520805, MT520806, MT708139, MT752653, MT752654, MT752657, MT752674, MT752676, MT752677, MT752690, MT752691, MT752722, MT752723, MT752732, MT752733, MT752784, MT752813, MT752816, MT752818, MT752820, MT752828, MT752830, MT752834, MT752835, MT752839, MT752840, MT752866, MT752868, MT752872, MT752873, MT752886, MT752888, MT752889, MT752898, MT752899, MT752902, MT752905, MT752912, MT752913, NC_011620;

Cluster C (33)
KJ534604, MT752612, MT752617, MT752619, MT752620, MT752636, MT752637, MT752639, MT752640, MT752641, MT752642, MT752656, MT752684, MT752713, MT752716, MT752725, MT752728, MT752734, MT752741, MT752779, MT752782, MT752814, MT752815, MT752822, MT752824, MT752846, MT752855, MT752876, MT752920, MT752921, MT752922, MT752923, MT752929;

Cluster D (10)
MT752618, MT752621, MT752627, MT752635, MT752638, MT752643, MT752714, MT752751, MT752777, MT752885;

Cluster E (3)
MT708140, MT708138, MT708137;

Cluster F (33)
AF172259, KJ534602, KJ534605, MT708141, MT708142, MT752663, MT752675, MT752710, MT752712, MT752727, MT752746, MT752769, MT752770, MT752772, MT752785, MT752788, MT752795, MT752801, MT752802, MT752804, MT752858, MT752870, MT752883, MT752884, MT752892, MT752900, MT752904, MT752907, MT752910, MT752918, MT752930, MT752936, X55802;

Cluster G (93)
KJ534601, KJ534603, MT752611, MT752613, MT752614, MT752616, MT752622, MT752624, MT752630, MT752647, MT752659, MT752660, MT752664, MT752665, MT752673, MT752678, MT752679, MT752680, MT752681, MT752682, MT752683, MT752686, MT752687, MT752688, MT752692, MT752694, MT752695, MT752696,

MT752698, MT752699, MT752700, MT752702, MT752704, MT752706, MT752708, MT752709, MT752726, MT752738, MT752740, MT752742, MT752744, MT752747, MT752748, MT752756, MT752760, MT752764, MT752765, MT752773, MT752774, MT752775, MT752776, MT752778, MT752780, MT752781, MT752789, MT752792, MT752796, MT752798, MT752800, MT752803, MT752805, MT752806, MT752807, MT752810, MT752812, MT752823, MT752832, MT752842, MT752859, MT752861, MT752862, MT752865, MT752867, MT752871, MT752874, MT752875, MT752881, MT752882, MT752887, MT752890, MT752891, MT752894, MT752895, MT752897, MT752901, MT752906, MT752908, MT752914, MT752916, MT752926, MT752928, MT752931, MT752932;

Cluster H (8)
MT752623, MT752661, MT752666, MT752667, MT752819, MT752848, MT752854, MT752860;

Cluster I (5)
MT752625, MT752693, MT752817, MT752825, MT752829;

Cluster J (4)
MT752766, MT752809, MT752811, MT752838;

Cluster K (5)
MT752745, MT752757, MT752767, MT752768, MT752799;

Cluster L (4)
MT752754, MT752790, MT752793, MT752794;
